# Highly Conserved Interaction Profiles between Clinically Relevant Mutants of the Cytomegalovirus CDK-like Kinase pUL97 and Human Cyclins: Functional Significance of Cyclin H

**DOI:** 10.3390/ijms231911814

**Published:** 2022-10-05

**Authors:** Martin Schütz, Regina Müller, Eileen Socher, Christina Wangen, Florian Full, Emanuel Wyler, Diana Wong, Myriam Scherer, Thomas Stamminger, Sunwen Chou, William D. Rawlinson, Stuart T. Hamilton, Heinrich Sticht, Manfred Marschall

**Affiliations:** 1 Institute for Clinical and Molecular Virology, FAU Erlangen-Nürnberg, 91054 Erlangen, Germany; 2 Division of Bioinformatics, Institute of Biochemistry, FAU Erlangen-Nürnberg, 91054 Erlangen, Germany; 3 Functional and Clinical Anatomy, Institute of Anatomy, FAU Erlangen-Nürnberg, 91054 Erlangen, Germany; 4 Institute of Virology, University Medical Center, Faculty of Medicine, Albert-Ludwig-University Freiburg, 79110 Freiburg, Germany; 5 Max-Delbrück-Center for Molecular Medicine (MDC), 13125 Berlin, Germany; 6 Serology and Virology Division, NSW Health Pathology, Prince of Wales Hospital, Sydney 2031, Australia; 7 School of Medical Sciences, University of New South Wales, Sydney 2052, Australia; 8 Institute for Virology, Ulm University Medical Center, 89070 Ulm, Germany; 9 Division of Infectious Diseases, Oregon Health and Science University, Portland, OR 97239, USA; 10 Department of Veterans Affairs Medical Center, Portland, OR 97239, USA; 11 School of Women’s and Children’s Health, University of New South Wales, Sydney 2031, Australia; 12 School of Biotechnology and Biomolecular Sciences, University of New South Wales, Sydney 2033, Australia

**Keywords:** human cytomegalovirus, viral CDK-like kinase, pUL97/vCDK, human cyclin complexes, pUL97–cyclin interaction, functional relevance, clinically relevant viral mutants, kinase activity, mapping and knock-out analyses, cyclin H functional significance, viral replication efficiency

## Abstract

The complex host interaction network of human cytomegalovirus (HCMV) involves the regulatory protein kinase pUL97, which represents a viral cyclin-dependent kinase (CDK) ortholog. pUL97 interacts with the three human cyclin types T1, H, and B1, whereby the binding region of cyclin T1 and the pUL97 oligomerization region were both assigned to amino acids 231-280. We further addressed the question of whether HCMVs harboring mutations in ORF-UL97, i.e., short deletions or resistance-conferring point mutations, are affected in the interaction with human cyclins and viral replication. To this end, clinically relevant UL97 drug-resistance-conferring mutants were analyzed by whole-genome sequencing and used for genetic marker transfer experiments. The recombinant HCMVs indicated conservation of pUL97–cyclin interaction, since all viral UL97 point mutants continued to interact with the analyzed cyclin types and exerted wild-type-like replication fitness. In comparison, recombinant HCMVs UL97 Δ231-280 and also the smaller deletion Δ236-275, but not Δ241-270, lost interaction with cyclins T1 and H, showed impaired replication efficiency, and also exhibited reduced kinase activity. Moreover, a cellular knock-out of cyclins B1 or T1 did not alter HCMV replication phenotypes or pUL97 kinase activity, possibly indicating alternative, compensatory pUL97–cyclin interactions. In contrast, however, cyclin H knock-out, similar to virus deletion mutants in the pUL97–cyclin H binding region, exhibited strong defective phenotypes of HCMV replication, as supported by reduced pUL97 kinase activity in a cyclin H-dependent coexpression setting. Thus, cyclin H proved to be a very relevant determinant of pUL97 kinase activity and viral replication efficiency. As a conclusion, the results provide evidence for the functional importance of pUL97–cyclin interaction. High selective pressure on the formation of pUL97–cyclin complexes was identified by the use of clinically relevant mutants.

## 1. Introduction

Human cytomegalovirus (HCMV) is a globally distributed β-subfamily member of *Herpesviridae*. HCMV infection causes life-long latency in the human host. In immunocompetent individuals, HCMV may remain asymptomatic, whereas in the immunosuppressed or immunonaïve host, such as transplant recipients, tumor patients, AIDS patients, as well as newborns, HCMV infection can lead to severe symptoms and life-threatening viral pathologies [1,2,3]. Most seriously, congenital HCMV infection can lead to a severe clinical phenotype in newborns, with long-term neurodevelopmental and audiological sequelae in infancy [4,5,6]. Viral pathogenesis is closely linked to the efficiency of viral replication in individual tissues. In this context, the role of host factors, especially the interaction through virus–host multiprotein complexes, needs to be deciphered in greater detail [7,8,9,10,11].

Notably, HCMV replication drastically interferes with cell-cycle regulation, a process in which the HCMV-encoded protein kinase pUL97 extensively phosphorylates the checkpoint regulator retinoblastoma protein (Rb) [12,13,14]. This initial Rb inactivation, followed by further viral regulatory intervention steps, ultimately results in early S-phase cell-cycle arrest accompanied by significant dysregulation of cyclin-dependent kinases (CDKs) and cyclins, termed as pseudomitosis [11,15]. Interestingly, HCMV encodes the serine–threonine protein kinase pUL97 that represents a viral CDK ortholog (vCDK) combining typical structural and functional features of host CDKs [10,12,16,17,18]. Previously, the current authors and others identified a specific feature of pUL97, in that it associates with human cyclins of various types, most abundantly with T1, H, and B1, according to our investigations [10,11,19,20,21,22,23,24]. Based on these observations, pUL97 is considered as a multiple cyclin-binding kinase, so that in addition to the types T1, B1, and H, further cyclin-binding activities, e.g., including cyclin A, might play supplementary roles [11,18,20,25]. In general terms, cyclins are a large family with 29 proteins in humans, structurally defined by the presence of the so-called cyclin box, a domain of approximately 100 amino acid residues that forms α-helices and provides an interface for binding to CDKs. Their intracellular concentrations vary cyclically during the cell cycle, and these oscillations, specifically fluctuations in gene expression of cyclins, then induce changes in CDK activity driving the cell cycle [25,26]. Notably, the cell cycle-related CDKs are typically characterized by multiple cyclin binding, whereas transcriptional CDKs are usually single cyclin binding. Formation of the CDK–cyclin complex results in activation of the CDK active site, and complete activation requires distinct regulatory steps of site-specific phosphorylation. The pUL97–cyclin B1 interaction was found to be phosphorylation-dependent for both proteins, suggesting that cyclin B1 binding and modulation through pUL97 might be linked to HCMV-induced alteration of the cell cycle and host CDK–cyclin machinery [22,24], although this awaits further experimental confirmation. The role of cyclin types T1 and H has been assigned to a bridging function of pUL97–pUL97 dimerization and oligomerization, and the formation of a putative quaternary complex [10,11,20]. In this regard, it is important to note that the minimal binding regions responsible for pUL97–cyclin T1 interaction and pUL97–pUL97 oligomerization showed a complete overlap in N-terminal amino acids 231-280. Studies involving viral genomic deletion mutants in the ORF-UL97 region clearly indicated that this region determines not only cyclin T1 interaction, but also cyclin H interaction, and that these interactions are functionally required for efficient viral replication [10].

The detailed structure of HCMV pUL97 has not yet been deciphered, but molecular modeling based on available CDK crystal structures has provided important information [22,24,27,28,29,30]. Notably, pUL97 consists of a widely unstructured N-terminal portion, including two classical nuclear localization signals (NLS1 and NLS2), and a large globular domain in its C-terminal half [11]. According to the results of previous investigations, these two regional blocks of pUL97 did not show biochemically relevant overlaps, neither in structural nor functional aspects. However, comparing the binding situation between the three human cyclin types preferentially binding to pUL97 [11,20,22], i.e., T1, H, and B1, the computational approach suggested that cyclin T1 and H may bind to parts of the pUL97 N-lobe, while B1 binding may involve N- and C-lobes [23]. More specifically, we identified in our previous investigations two main candidate regions in pUL97 that represent determinants of cyclin interaction, i.e., the abovementioned linear binding motif contained within the unstructured N-terminal pUL97 region 231-280, termed interface 2 (IF2, amino acids 231-280) [10], and a larger contact area within the C-terminal globular kinase region termed IF1. In the case of cyclin T1 binding, this larger interface IF1 was not by itself able to confer a stable pUL97–cyclin interaction, but was dependent on the presence of the dominant IF2 [10]. The deletion of IF2 (Δ231-280) led to the loss of pUL97 interaction with cyclins T1 and H (but not B1), thus emphasizing its dominant binding-determining role within the N-terminal region. Interestingly, in the case of cyclin B1, the C-terminal region harboring the pUL97 kinase domain also has an impact on pUL97–cyclin interaction. Our previous data clearly indicated that the kinase-null mutation K355M as well as short deletions in the extreme C-terminus are crucial for activity, and pUL97-inhibitory small molecules completely abrogated cyclin B1 interaction [24]. This finding is compatible with our structural model predicting that at least part of the globular kinase domain contributes to the cyclin binding interface [23]. A second interesting aspect of this C-terminal region of pUL97, in particular amino acids 337-651, is given by the fact that the fragment is typically responsible for formation of pUL97-conferred drug resistance, i.e., ganciclovir (GCV) or maribavir (MBV) resistance [11,16]. Thus, the question arose whether this has additional importance for cyclin binding, i.e., whether the N-terminal and C-terminal regions of pUL97 may possess a codetermining impact on the binding of the three analyzed types of cyclin. In the present study, we build on this information about virus-supportive protein complexes between pUL97 and cyclins, and provide novel data that demonstrate the conservation of this interaction and its functionality within clinically relevant mutants of HCMV.

## 2. Results

### 2.1. Whole Genome Sequencing of Drug-Resistant Clinical Isolates of HCMV

Recently, several studies have reported on HCMV sequence information derived from comprehensive analyses using clinically relevant HCMVs as well as reference and laboratory strains [31,32,33,34,35,36,37]. In order to collect more information about strain sequence variation of isolates in our present study, we performed viral whole genome sequencing (WGS) and comparative analysis of clinical isolates of HCMV. To this end, we applied the Agilent SureSelectXT system to reveal the WGS of ten drug-resistant HCMVs. All HCMV isolates showed mainly cell-bound replication characteristics, so we directly used the total cellular DNA fractions of low passage numbers of HCMV-infected primary human foreskin fibroblasts (HFFs). As an important result, we resolved the ORF-UL97 primary sequences as well as those of viral pUL97 kinase substrate proteins and all additional genomic sequence information (Table 1).

**Figure 1:**
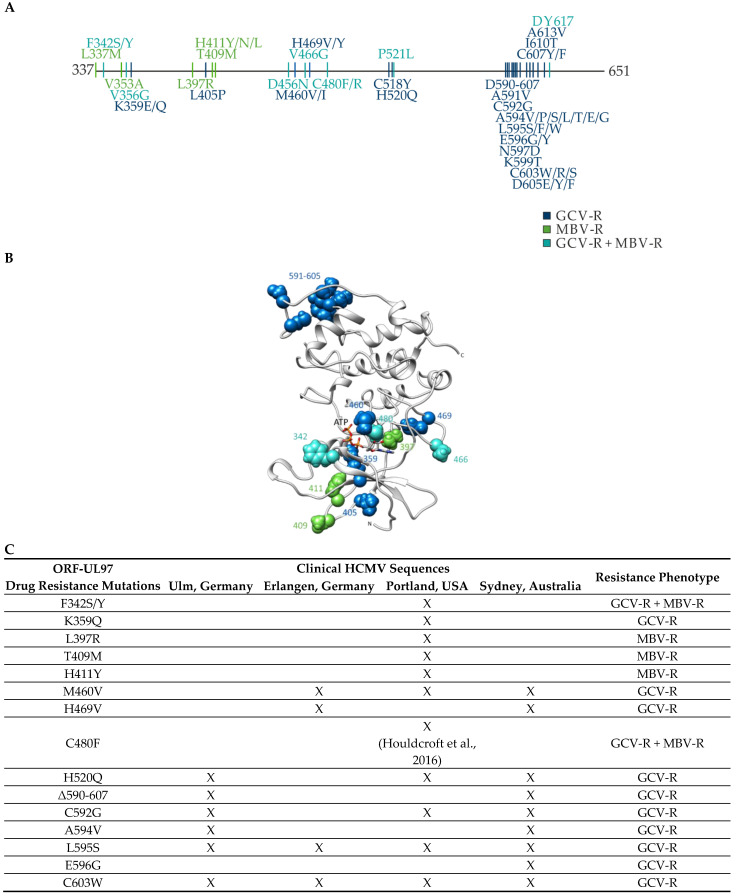
Overview of identified and previously known drug resistance mutations in ORF-UL97. (**A**) HCMV ORF-UL97 drug resistance mutations detected in clinical specimens or cell culture. A schematic map shows resistance-conferring mutations in the globular domain (amino acids 337-707) that were analyzed in this study [16]. The resistance phenotype is color-marked. (**B**) Predicted structural allocation of pUL97 drug resistance mutations. The location of drug resistance mutations in the predicted structure of the pUL97 C-terminal region shows an accumulation in positions 342-480 around the ATP-binding site of the active center. Note that the sites of GCV-R and MBV-R mutations are not clearly separated in their structural locations (color code of resistance site identical with panel A). (**C**) The tabular view shows the resistance mutations sequenced in clinical isolates that were transferred into the TB40-IE2-YFP standard genome for further analysis in this study. The designation of clinical HCMV sequences refers to: Ulm, Germany, laboratory of T.S.; Portland, USA, laboratory of S.C.; Sydney, Australia, laboratory of W.D.R.; Erlangen, Germany, laboratory of M.M. Resistance mutation C480F was first sequenced by Houldcroft et al., 2016 [39].

Here, we focused on ORF-UL97 single-site mutations, i.e., amino acid replacement mutations and short deletions, conferring resistance to GCV or MBV, or double resistance (GCV-R + MBV-R). The identified and previously known drug resistance mutations in ORF-UL97 are summarized by a comparative overview (Figure 1A). Notably, the set of drug-resistance mutations detected either in clinical specimens or in cell-culture-based infection models is contained within the C-terminal half of pUL97, amino acids 337-651 [16]. A distinctive feature was the accumulation of MBV-R mutations (green) in the N-terminal stretch of this fragment, whereas GCV-R mutations were more frequent in the C-terminal stretch (blue, mostly amino acids 590-617), and double-resistance mutations were found to be spread out (cyan). As seen from the structural view (i.e., based on a CDK-derived predicted model structure), resistance-conferring mutations were mainly found accumulated in positions 342-480 around the ATP-binding site of the active center (Figure 1B). Thereby, the structural locations of GCV-R and MBV-R mutations were not clearly distinguished between separate structural elements, but were seen superimposed in the N-terminal half of the globular structure (residues 520 and 521 were not resolved in the model). This was also illustrated by the fact that GCV-R + MBV-R double-resistance mutations were found in the same area. The GCV-R mutations assigned to the C-terminal half, however, were located at some distance from the active center of the kinase domain, so their mechanistic role in the determination of drug resistance might be complex. Given these points of information, a series of 29 clinical HCMV sequences was collected from four different laboratories, eventually covering a broad variety of available ORF-UL97 drug resistance mutations (Figure 1C). Interestingly, among the 15 different genotypes of drug resistance, five mutations were found at high frequency, i.e., M460V, H520Q, C592G, L595S and C603W, in three or four of the isolates. This collection was then used for generating a representative series of reporter viruses carrying resistance mutations. For this purpose, 15 selected ORF-UL97 resistance mutations (Figure 1C) were transferred into the HCMV TB40-IE2-YFP reporter genome published previously [38].

### 2.2. ORF-UL97 Sequence-Specific Analysis of HCMV Isolates

Since no 3D structure of HCMV pUL97 has so far been resolved, molecular modeling was employed to provide a structural interpretation of the experimental data. Using this approach, two different interfaces were characterized to confer cyclin binding: (i) the interface between the 50 N-terminal amino acids of pUL97 (231-280, IF2) and cyclin T1, and (ii) a secondary binding interface contained within the globular C-terminus of pUL97, which is considered to contribute additional cyclin-binding activity (IF1). This C-terminal region of pUL97, spanning amino acids 337-651, is in all cases responsible for typical pUL97-conferred drug resistance formation, i.e., GCV or MBV resistance. Against this background information, we speculated whether these two regions of interest, i.e., 231-280 (IF2) and 328-655 (harboring the poorly defined IF1 of cyclin binding, and with importance for drug resistance) may possess greater potential to influence each other than initially expected. Specifically, the question was addressed whether drug resistance mutations detected within the C-terminal region, 328-655, may have an impact on the candidate region, 231-280, of cyclin binding. For a first analysis of the natural sequence variability in these regions on the basis of their primary sequences, we used 473 GenBank-annotated ORFs-UL97 of various HCMVs (accession numbers are given by Table S1). When comparing the probability of mean exchange of amino acids between given regions of the 473 ORFs-UL97, we calculated a low value of 0.66‰ for the C-terminal globular domain 328–655, but a higher value of 2.01‰ for the non-structured N-terminal region 1–327. This result differed,, however, when specifically focusing on the group of 43 (out of 473) ORFs-UL97 that carryied UL97 replacement markers of drug resistance, i.e., 3.83‰ C-terminal compared to 1.85‰ N-terminal. Thus, the selection pressure of mutations conferring drug resistance led to an increase of exchange probability in the C-terminal globular domain. This situation was almost identical for our group of 10 newly sequenced drug-resistant HCMV mutants (Ulm), i.e., 3.36‰ C-terminal compared to 1.53‰ N-terminal. Interestingly, when specifically addressing the question of replacement mutations contained within the N-terminal region of 231–280, relevant to cyclin-binding, we observed a very low exchange probability of 0.00‰ in the case of the 10 newly sequenced mutants. Although we were aware of the limitation of such a basic calculation, which may be taken to indicate a preliminary tendency, the point prompted us to investigate further the importance of these ORF-UL97 regions for cyclin binding, by performing functional studies.

### 2.3. Resistance-Conferring Mutations of ORF-UL97 in Virus Recombinants Only Show a Minor Impact on Viral Replication Fitness

**Figure 2:**
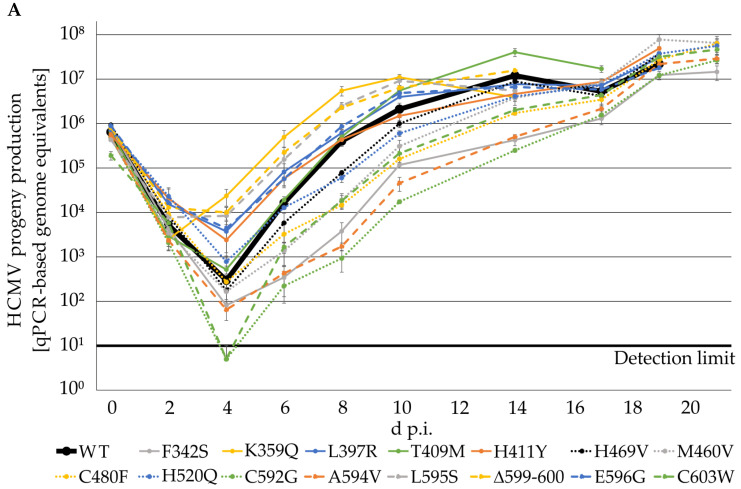

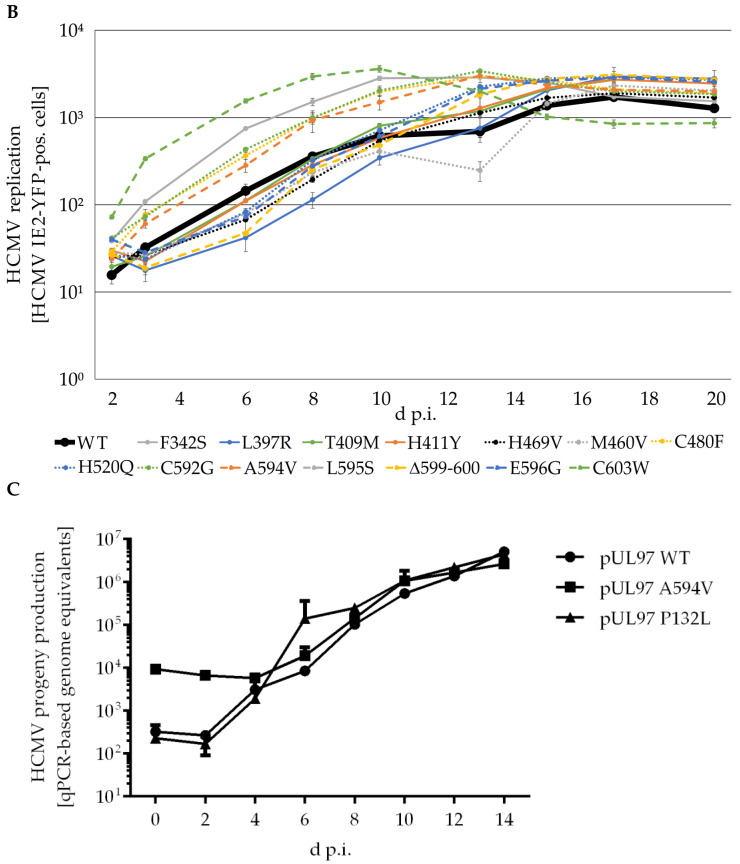
Viral replication kinetics determined by HCMV-specific qPCR and quantitation of IE2-YFP positive cells. (**A**) 225,000 HFFs in 12-well plates were infected with parental HCMV strain TB40 WT or ORF-UL97 point mutants at a viral dose of 1 × 10^5^ genome copies. Viral supernatants were harvested at indicated time points and viral genome equivalents were determined by qPCR. Each value represents the mean ± SD of two independent biological replicates, each measured twice. (**B**) Alternatively, 13,500 HFFs in 96-well plates were infected at an MOI of 0.01 and the number of HCMV-infected cells was measured by detection of YFP signal-positive cells at indicated time points, using ImageXpress Pico. Values are given as mean value ± SD of three independently infected wells. (**C**) HFFs were infected with parental HCMV strain Merlin WT or ORF-UL97 point mutations (A594V, conferring GCV resistance; P132L, not conferring drug resistance) at an MOI of 0.001. Viral genome copies in the supernatant were determined using qPCR. Each value is given as the mean ± SD of three independent biological replicates.

A specific selection of 15 drug-resistance-conferring markers were used for genetic transfer into the yellow fluorescence protein (YFP)-expressing reporter virus of HCMV reference strain TB40, using a traceless Red recombination system [10,39,40]. The newly generated stock viruses of these mutants (Figure 1C) were used for comparing the kinetics of viral replication between the recombinant HCMVs and the parental wild type (WT/TB40-IE2-YFP). To this end, HFFs were infected with parental HCMV strain TB40 WT or ORF-UL97 replacement mutants at a viral dose of 1 × 10^5^ genome copies, and viral genome equivalents derived from supernatants were measured via qPCR. The qPCR-based analysis of progeny virus released into the culture supernatants of the infected HFFs resulted in a mostly homogeneous pattern of replication curves (Figure 2A). Some of these mutants, such as C592G, T409M, A594V, F342S, and others, showed some delay in reaching WT-like levels of progeny production, while other mutants such as K359Q, C480F, L595S, L397R, and others showed slightly accelerated kinetics. Several mutants were almost indistinguishable from the WT, such as H469V, T409M, H520Q, H411Y, and others. Importantly, during the final phase of investigation between 17 to 21 days post-infection (d.p.i.), all viral mutants basically attained WT-like quantities of virus production. Furthermore, the slopes of the growth curves of the initially delayed mutants were largely identical in the exponential phase compared to WT. An imaging-based quantitative measurement of HCMV IE2-YFP-positive cells provided a confirmation of the described replication characteristics of the mutants (Figure 2B). Moreover, the analysis was extended to include comparison with another viral strain, HCMV Merlin, using BACmid-derived recombinants expressing either pUL97 WT, mutant A594V (GCV resistance), or mutant P132L (no drug resistance). Contrary to our initial hypothesis that individual HCMV replacement mutants in pUL97 might possibly differ significantly in their quantitative levels of replicative fitness, we detected no specific differences. Furthermore, the two Merlin mutants, which both carried pUL97 amino acid replacements outside region 231-280 (IF2 of cyclin binding), showed very similar replication kinetics (Figure 2C). This supports our current findings that strains and ORF-UL97 variants of HCMV, including those carrying prominent mutations such as A594V and P132L, are not clearly distinguishable by quantitative readouts. In combination, the results indicate no effect or only minor impact of drug-resistance-conferring ORF-UL97 mutations on viral replication efficiency.

### 2.4. Interaction Profiles Show a High Conservation of pUL97–Cyclin Complexes for Clinically Relevant Point Mutants in HCMV Strain Backgrounds TB40 and AD169

**Figure 3:**
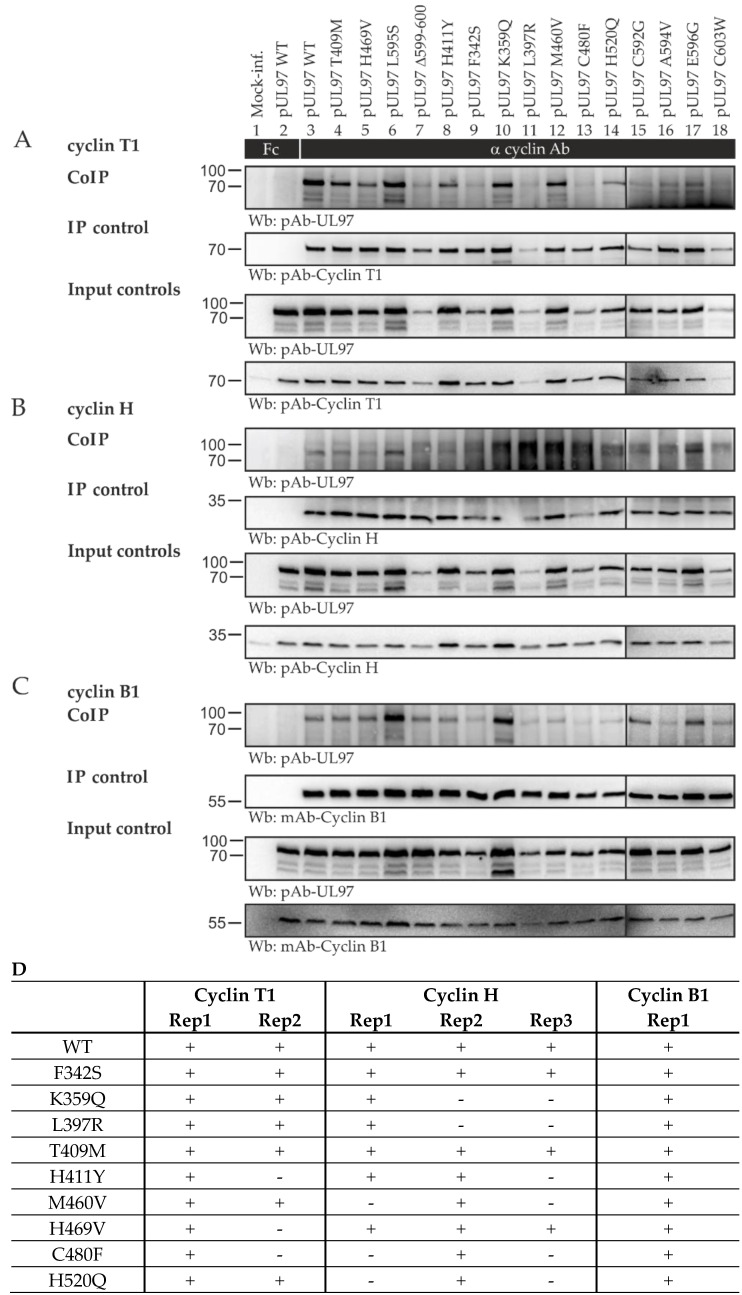

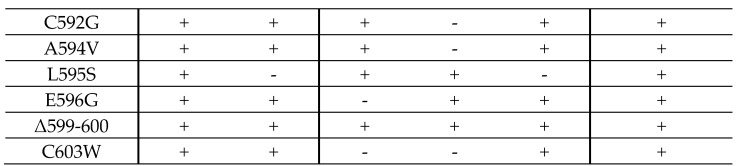
Interaction analysis of TB40-IE2-YFP ORF-UL97 resistance mutants with human cyclins. HFFs in T75 flasks were infected with HCMV TB40-IE2-YFP WT or newly generated ORF-UL97 point mutants. Cells were harvested between 4 and 7 d.p.i., when approximately 80% of the cells showed a cytopathic effect. Cells were lysed, and human cyclins T1 (**A**), H (**B**), and B1 (**C**) were immunoprecipitated using specific antibodies. A chicken Fc fragment served as specificity control, and lysate taken prior to immunoprecipitation was used as input control. All samples were subjected to Wb analysis, and proteins were detected using specific monoclonal and polyclonal antibodies. (**D**) A summary is given of biological replicates (Rep1, Rep2, Rep3) monitoring the interaction of pUL97 mutants with human cyclins T1, H, and B1.

The recombinant HCMVs generated in this study were then analyzed for their properties relating to pUL97–cyclin interaction. Since earlier studies performed by our group highlighted that the most abundant interactions of the pUL97 kinase were with host cyclin types T1, H, and B1, the focus was directed onto these detectable complexes. Specifically, we addressed the question whether one or several of the mutants may have lost individual interaction properties. The signal pattern of this coimmunoprecipitation (CoIP) analysis clearly showed some quantitative variation in the detectable immunoprecipitated cyclins (Figure 3), whereby the levels of pUL97 expression varied similarly (see panels of input control) but the actual amounts of coimmunoprecipitated pUL97 were constant (see panels of CoIP). As an important result, all mutants in different independent replicates showed at least a basic level of interaction with these three cyclins (Figure 3A–C), while the quantitative variations did not show a clear correlation with individual sites of mutation (Figure 3D). Thus, entirely defective cyclin interaction was not observed for any of the pUL97 mutants. The obtained picture suggested the presence of variations, at least in this CoIP approach, but indicated no lack of pUL97–cyclin interaction in any of these cases.

**Figure 4:**
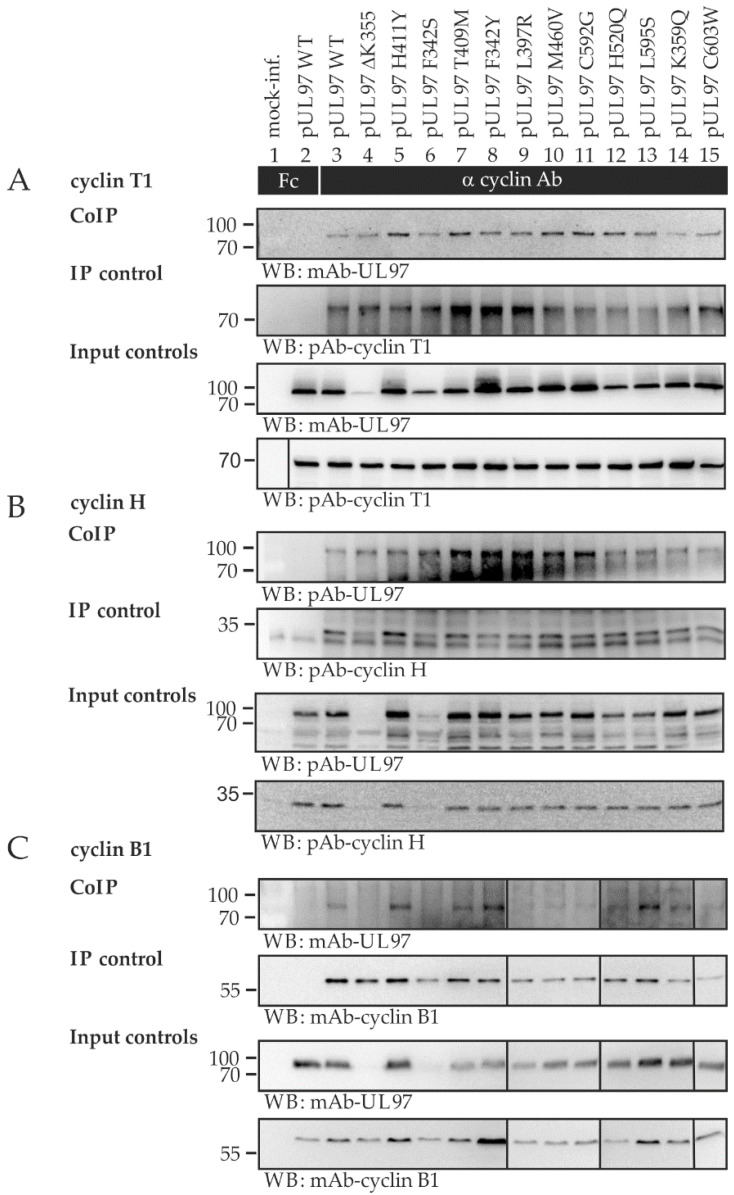
Interaction analysis of AD169 pUL97 resistance mutants with human cyclins. HFFs in T75 flasks were infected with HCMV AD169 WT or UL97 point mutants (UL97 WT and mutants were derived from Sunwen Chou, Portland, OR, USA). Cells were harvested between 4 and 7 d.p.i., when approx. 80% of the cells showed a cytopathic effect. Cells were lysed, and human cyclins T1 (**A**), H (**B**), and B1 (**C**) were immunoprecipitated using specific antibodies. A chicken Fc fragment served as specificity control, and lysate taken prior to immunoprecipitation was used as input control. All samples were subjected to Wb analysis, and proteins were detected using the specific monoclonal and polyclonal antibodies indicated.

The question of constancy or clear-cut differences in cyclin interaction was investigated using a similar series of mutants with the genetic background of the HCMV strain AD169, published earlier. The possibility of strain-specific differences in the pUL97–cyclin interaction patterns appeared plausible on the basis that strains of HCMV show slight differences in pseudomitosis regulation of the host CDK–cyclin machinery [15]. The CoIP results obtained were very similar to those described above for strain TB40 (Figure 4). Furthermore, a quantitative variation in pUL97 expression levels and coimmunoprecipitated pUL97 was detected in this case, while the amounts of immunoprecipitated cyclins remained more constant. Although signal levels recorded by the CoIP approach were low for individual cyclins, no lack of pUL97–cyclin interaction could be confirmed. As a conclusion, quantitative variation in the formation of these virus–host protein complexes is evident, and these findings might indicate selection pressure preserving the pUL97 interactions, thus pointing to their general functional importance.

### 2.5. Amino Acid Region 231-280 of ORF-UL97 Strictly Restricts Cyclin T1/H Interactions and Viral Replication Efficiency

**Figure 5:**
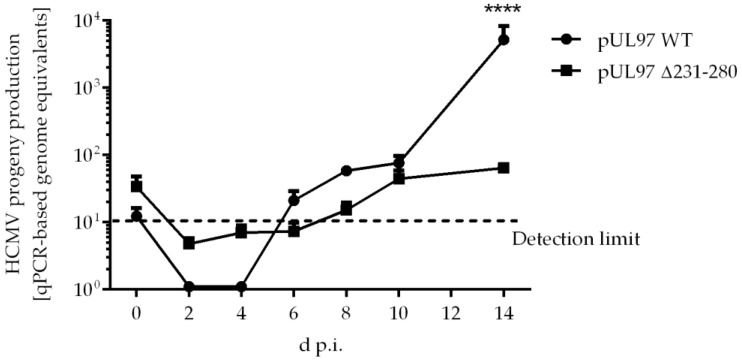
Viral replication kinetics of pUL97 WT and pUL97 Δ231-280 determined by HCMV-specific qPCR. In total, 225,000 HFFs in 12-well plates were infected with parental HCMV strain AD169-GFP WT or AD169-GFP pUL97 Δ231-280 at an MOI of 0.001. Viral supernatants were harvested at indicated time points and viral genome equivalents were determined by qPCR. Each value represents the mean ± SD of two independent biological replicates, each measured twice. Statistical analysis was performed using an ordinary two-way ANOVA and post-hoc Sidak correction; ****, *p* < 0.0001.

As a main finding of our recent report, we identified a profound phenotypical defect of a recombinant HCMV carrying a small deletion in ORF-UL97, Δ231-280 [10]. The causative regulatory issue concerning this viral phenotype was assigned to a lack of the interaction between pUL97 and cyclins T1 and H. Several levels of consequences for pUL97 functionality have since been experimentally addressed, referring to affected kinase activity. This kinase defect was characterized by a strong impairment of interaction and catalytic properties, i.e., self-interaction and autophosphorylation as well as substrate interaction and phosphorylation were found impaired in comparison with the wildtype virus (WT). Here, the Δ231-280 virus showed a strong reduction in viral replication with a very limited increase in viral genome equivalents over the analyzed timeline (Figure 5). In consequence, this qPCR-based quantitation of HCMV genome equivalents in HFFs showed a statistically highly significant reduction in the production and release of pUL97 Δ231-280 progeny virus compared with WT at 14 d.p.i.. To further characterize this defective region, additional HCMV deletion mutants were generated, using WT as the parent for further recombinations.

**Figure 6:**
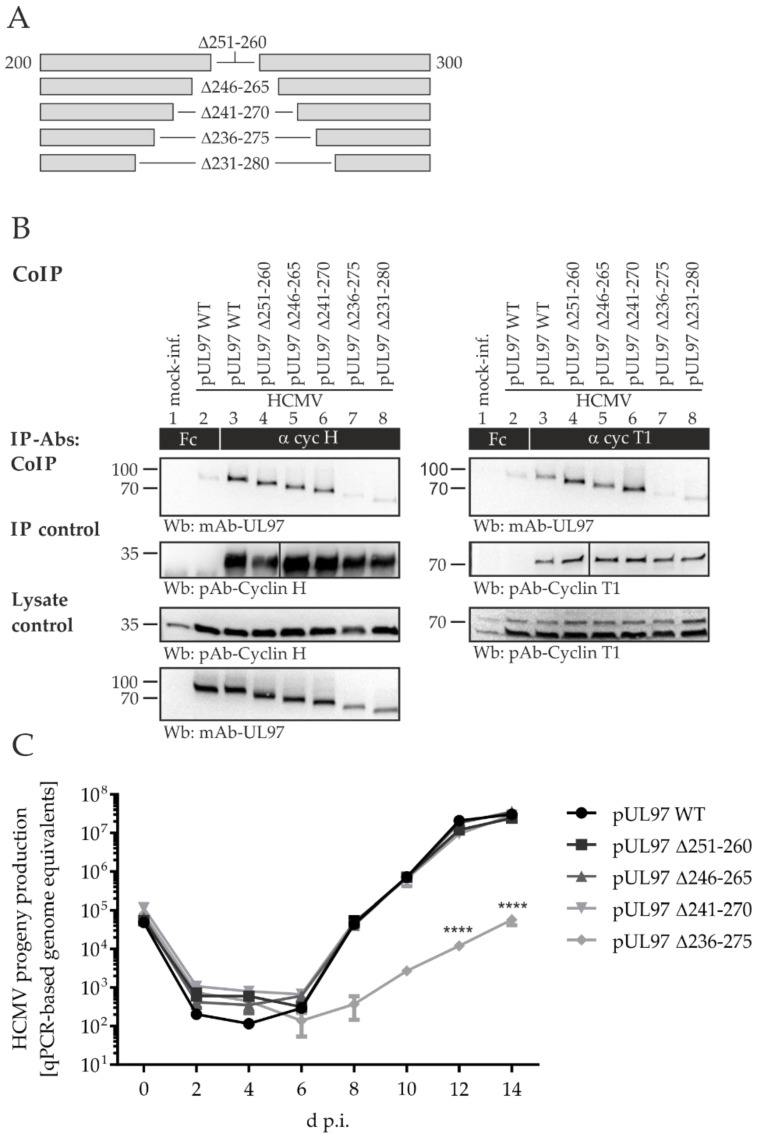
Interaction analysis with human cyclins, and viral replication kinetics of HCMV ORF-UL97 deletion mutants within the amino acid region 231-280. (**A**) A schematic depiction of newly generated HCMV ORF-UL97 deletion mutants in fragments of amino acids 231-280. (**B**) HFFs in T175 flasks were infected with HCMV AD169 WT, ORF-UL97 mutant versions, or remained mock-infected. Cells were harvested and lysed 4 d.p.i. Lysate control samples were taken before human cyclins H and T1 were immunoprecipitated using the specific antibodies indicated. A chicken Fc fragment served as negative control. All samples were subjected to Wb analysis, and proteins were detected using specific monoclonal and polyclonal antibodies as indicated below the panels. (**C**) A total of 225,000 HFFs in 12-well plates were infected with parental HCMV strain AD169-GFP WT or ORF-UL97 deletion mutants at a viral dose of 1 × 10^5^ genome copies. Viral supernatants were harvested at indicated time points and viral genome equivalents were determined by qPCR. Each value represents the mean ± SD of two independent biological replicates, each measured twice. Statistical analysis was performed using an ordinary two-way ANOVA and post-hoc Sidak correction; ****, *p* < 0.0001.

In a next step, we generated four more deletion mutants within the amino acid region 231-280 of ORF-UL97, to define the minimal interaction fragment with human cyclins H and T1. We first deleted 10 amino acids from the center of the pUL97–cyclin binding region (Δ251-260) and then further extended the deletions by five more amino acids both up- and downstream (i.e., Δ246-265, Δ241-270, Δ236-275; Figure 6A). We then analyzed the pUL97–cyclin binding properties for the recombinant HCMVs, using CoIP analysis (Figure 6B). The signals detected in the lysate control samples showed mostly similar pUL97 protein levels (Figure 6B, lowest panels; note that in the cases of the two largest deletions, Δ236-275 and Δ231-280, a relative decrease of pUL97 levels was detected in each, a phenomenon we already observed for Δ231-280 in a previous study [10]). Importantly, the smaller pUL97 deletion mutants Δ251-260, Δ246-265, Δ241-270 preserved a WT-like, strong interaction with cyclin H and cyclin T1 (Figure 6B, lanes 3–6). In contrast, however, interaction with cyclin H and T1 was negative for the mutant Δ236-275 (lane 7), very similar to the largest deletion mutant Δ231-280 used as a reference (lane 8; compare the marginal levels of residual signals in lanes 7–8 with the background of the Fc negative control in lane 2). Thus, the minimal interaction fragment determined by this analysis was found to be defined by amino acids 236-275. We then analyzed the progeny virus release of the newly generated HCMV AD169-GFP ORF-UL97 deletion mutants, by qPCR (Figure 6C). The smaller pUL97 deletion mutants Δ251-260, Δ246-265, and Δ241-270 showed WT-like virus production without detectable differences in viral replication fitness. Strikingly, deletion of amino acids 236-275 led to a statistically highly significant replicative impairment, which was compatible with the loss of cyclin H and T1 interaction, similar to the respective characteristics published earlier for deletion mutant Δ231-280.

### 2.6. Comparing HCMV Replication Efficiencies in Cyclin Knock-Out (KO) Cell Populations: Cyclin H KO Cells Populations, but Not Cyclin B1 KO or T1 KO, Determine a Strict Limitation of Viral Replication

**Figure 7:**
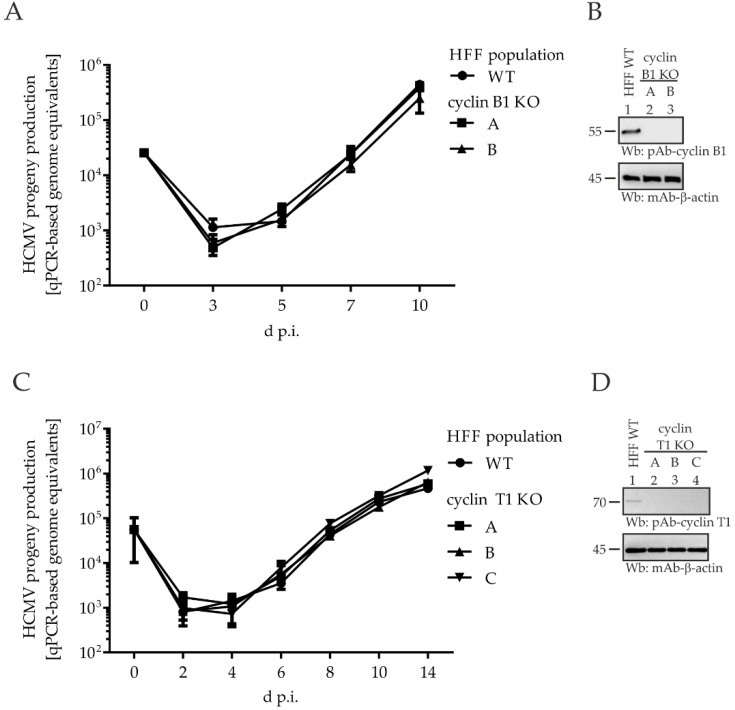
HCMV AD169-GFP replication kinetics in HFF WT and cyclin B1/T1 KO populations. HFFs were infected with HCMV AD169. Viral replication kinetics were determined by collecting viral supernatants at the indicated time points, and viral genome equivalents were determined by qPCR. Each value represents the mean ± SD of two independent biological replicates, each measured twice: (**A**) replication kinetics obtained for cyclin B1 KO cell populations A and B compared to WT (MOI 0.025); (**C**) replication kinetics obtained for cyclin T1 KO cell populations A, B and C compared to WT (MOI 0.01); (**B**,**D**) effectivity of cyclin B1 KO (cell populations A and B) and cyclin T1 KO (cell populations A, B and C), respectively, was verified by standard SDS-PAGE and Wb analysis using specific antibodies as indicated.

To address specifically the functional importance of viral cyclin interactions, we generated HFF cyclin KO cells using the CRISPR/Cas9 system, and used these cell populations for qPCR-based HCMV replication kinetics. Regarding the KO effects of cyclin B1 (Figure 7A) and T1 (Figure 7C), HCMV showed a WT-like replication efficiency, i.e., no impairment of the release of progeny virus could be detected. The efficiency of the KO was verified by Wb analysis, and no residual cyclin expression could be detected at the protein level, indicating the required stringency of cellular KO for cyclins B1 or T1, respectively (Figure 7B,D). In line with these findings, the investigation of viral protein expression throughout the course of infection, as monitored by Wb analysis, revealed no notable differences between cyclin B1/T1 KO and WT cell populations. Furthermore, in vitro analysis of viral kinase activity using a pUL97-specific kinase assay did not point to KO-mediated changes. Thus, these data led to the conclusion that the lack of neither cyclin B1 nor T1 exerts a decisive regulatory impact on pUL97, at least not detectable by these readouts under the described KO conditions.

**Figure 8:**
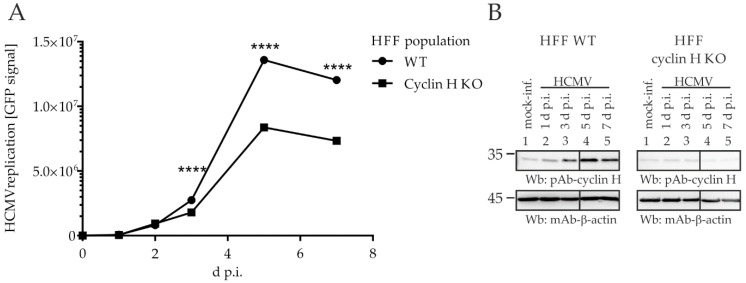
HCMV AD169-GFP replication kinetics in HFF WT and in transiently transduced HFFs with a partial level of cyclin H KO. A total of 225,000 HFFs were seeded in 12-well plates. The next day, cells were incubated with lentiviral supernatant for 24 h, introducing the CRISPR/Cas9 system and a cocktail of cyclin H-specific gRNAs for transient transduction. Subsequently, cells were incubated with fresh medium for 24 h. Next, HFF WT and cyclin H KO populations were infected with HCMV AD169-GFP at an MOI of 0.5 and harvested at the indicated time points. (**A**) Cells were lysed and the GFP signal of lysates was measured in triplicate using a Victor multilabel reader. Statistical analysis was performed using an ordinary two-way ANOVA and post-hoc Sidak correction; ****, *p* < 0.0001. (**B**) For comparison of intracellular cyclin H expression levels, the total cellular lysates used for GFP signal quantitation were subsequently denatured at the indicated time points and used for standard SDS-PAGE and Wb analysis. Cyclin H and β-actin were detected using specific antibodies as indicated.

Next, cyclin H was taken into consideration. With the aim of generating a cyclin H KO cell population, however, we were not successful as we had been for cyclin B1 and T1 KO. Using the identical conditions of genetic KO and cell selection, no stable cell population could be achieved that lacked or even showed markedly reduced cyclin H expression. This might have been due to the multifaceted importance of cyclin H–CDK7 complexes that affect cell cycle, transcription, and other regulatory features [40,41]. To overcome this limitation, we transiently transduced HFFs with the lentiviral particle transduction system containing the CRISPR/Cas9 activity and a cocktail of different cyclin H-specific gRNAs. This approach led to successful transient KO of non-selected HFFs, which were freshly used for subsequent experiments (Figure 8). These HFFs showed partial levels of cyclin H KO (Figure 8B) and were used 48 h after transduction, for the determination of a GFP-based HCMV replication kinetic. The monitoring of cyclin H protein levels by Wb analysis revealed an upregulation of cyclin H in HCMV-infected HFF WT, starting as early as 1 d.p.i. (Figure 8B, left). In contrast, such upregulation could not be detected for the transient cyclin H KO cell population (Figure 8B, right). Here, cyclin H levels remained constant during the first 3 d.p.i., before cyclin H was further reduced almost to the detection limit at later time points. The low levels of cyclin H, partially downregulated in this way, strictly correlated with impaired HCMV replication efficiency (Figure 8A), as indicated by the statistically significant reduction of GFP reporter signals starting from 3 d.p.i. Interestingly, the period of strongest impairment of HCMV replication (5–7 d.p.i.) coincided with the time points that showed further decreases in cyclin H levels (compare Figure 8A with the right-hand side of Figure 8B, lanes 4–5). Overall, a 40% reduction in viral replication efficiency was observed for the cyclin H KO population compared to WT cells. Interestingly, cyclin H and cyclin T1 both interact with pUL97 in the region 231-280, but cyclin H KO exclusively shows functional importance, while cyclin T1 KO can be tolerated. The findings indicate that the pUL97 interaction with cyclin T1 did not produce measurable defects according to our analysis systems, whereas the lack of cyclin H clearly translated into a replicative impairment. Our data provided no indication of a putative mutual reinforcing effect between pUL97–cyclin T1 and pUL97–cyclin H interaction. Thus, an important conclusion from this experimental setting is that cyclin H represents a major determinant of the viral replication efficiency. However, at this point the question remained open whether the strong inhibition of viral replication under cyclin H KO conditions refers directly to pUL97–cyclin H interaction or is linked indirectly with cyclin H-depleted CDK7 deregulation. We previously showed that inhibition of CDK7 strongly inhibits HCMV replication, therefore representing a promising target for antiviral therapy [42,43]. To exclude the possibility that the growth defect induced by cyclin H KO is exclusively caused by indirect CDK7-mediated effects, we next investigated the primary impact of cyclin H on pUL97-specific kinase activity.

### 2.7. pUL97 Kinase Activity Is Increased upon co-Transfection of Cyclin H Using a Quantitative Sox Peptide-Based In Vitro Kinase Assay (qSox-IVKA)

**Figure 9:**
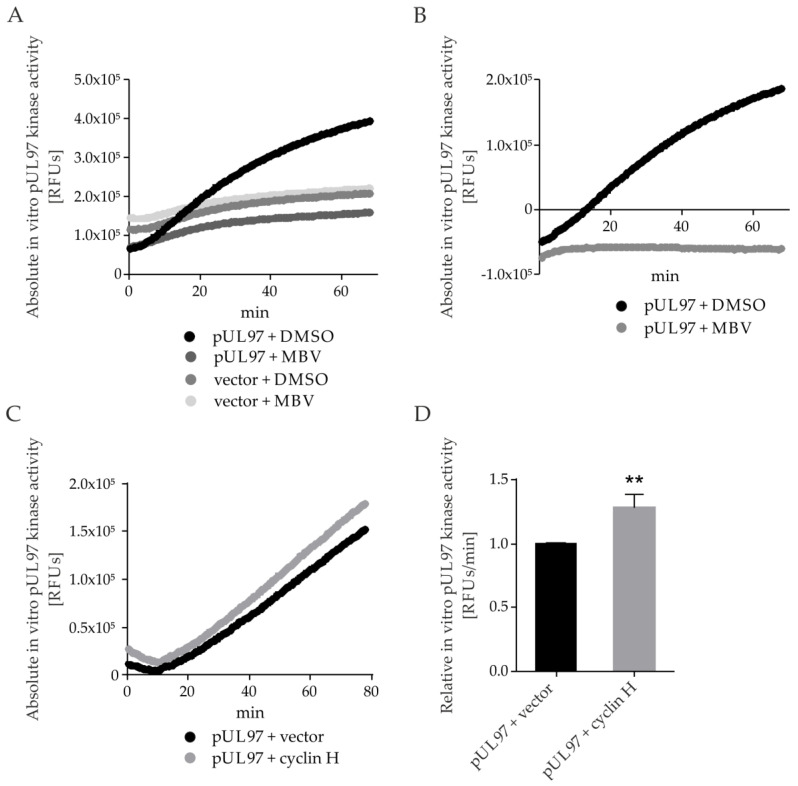

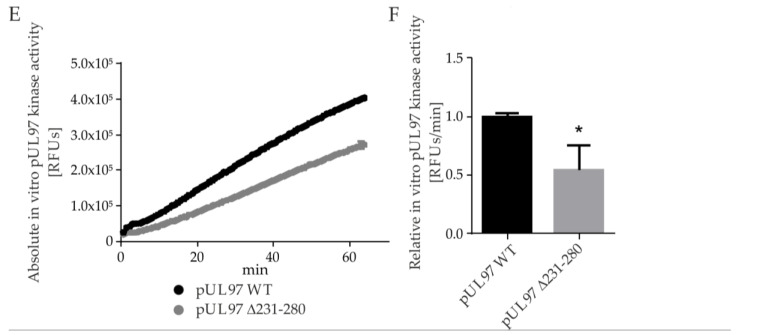
Application of the qSox-IVKA for quantitative measurement of in vitro pUL97 kinase activity. The 293T cells were seeded in 10-cm^2^ dishes for transfection on the following day using plasmids coding for pUL97-Flag or empty vector control. At 1 day post-transfection (d.p.t.), cells were lysed and pUL97-Flag was immunoprecipitated using 25 µL Dynabeads^®^ protein A coupled with Flag-tag specific antibodies. Dynabead-bound proteins were then eluted in 50 µL enzyme buffer and used for qSox-IVKA. For these reactions, 40 µL of kinase reaction mix was pipetted in Corning NBS 96-well half area microplates, before 10 µL Dynabeads protein solution was added. Kinase activity was measured using a Victor multilabel reader, at every 30 s of the kinetics for approximately 80 min. Values of pUL97 activity were normalized according to the expression levels on Wbs. (**A**) Absolute values of the measured RFUs for pUL97 and vector control, as treated with 5 µM of MBV or DMSO as the solvent control; measurements were performed in duplicate. (**B**) Empty vector controls from panel A were used for normalization of pUL97 signal intensities, to depict the specific activity. (**C**) The pUL97 kinase activity was determined by qSox-IVKA under conditions with or without cyclin H-HA coexpression (transient cotransfection setting); mean values of duplicates are given. (**D**) Relative pUL97 + cyclin H activity per min was calculated for the linear proportion of the curve obtained in panel C for 20–70 min, normalized to pUL97 + vector control. Statistical analysis was performed using an ordinary two-way ANOVA and post-hoc Sidak correction; **, *p* < 0.01. (**E**) Mutant kinase activity of construct pUL97 Δ231-280 was compared with pUL97 WT (both Flag-tagged), by qSox-IVKA. Mean values are given of two independent experiments, with measurements taken in duplicate. (**F**) Relative pUL97 activity per minute was calculated for the linear proportion of the curve obtained in panel E for 20–60 min, normalized to pUL97-Flag. Statistical analysis was performed using an ordinary two-way ANOVA and post-hoc Sidak correction; *, *p* < 0.05.

Our previous applications of pUL97-/CDK-specific in vitro kinase assays (IVKAs) were based on kinase–substrate CoIP, radioactive or nonradioactive ATP-specific labeling of phosphosites, and readouts by Wb analysis [10,22,44,45]. These assays, which were mostly suited for the identification of phosphorylated kinase substrates, had limitations in sensitivity and quantifiability. In this study, we were prompted to establish an improved pUL97-specific IVKA technology using a short peptide consisting of a distinct kinase recognition sequence, i.e., a phosphorylated serine or threonine target site, and a Sox amino acid. Phosphorylation of the serine or threonine residue enables Mg^2+^-binding between Sox and the newly introduced phosphate, whereby phosphorylation greatly enhances the affinity of the peptide for Mg^2+^. This chelation ultimately generates a fluorescence signal that can be detected by a multilabel plate reader under continuous measurement and can be used to monitor kinase activity over time, even under variable conditions [46,47]. An assay was performed to demonstrate the specificity of the peptide. Total lysates of transfected cells were used as the starting material, from which the ectopically produced pUL97 was immunoprecipitated using dynabeads and specific antibodies. Dynabead-bound pUL97, or empty beads derived from mock-transfected lysates, were utilized, and MBV was added as an optional pUL97-specific inhibitor control (Figure 9A; DMSO as solvent control). Signal intensities of the respective vector settings were subtracted from the pUL97-derived signals to obtain normalized pUL97-specific signal intensities (Figure 9B). A continuous increase in signal intensity was observed for pUL97 DMSO samples over the time course, in comparison to a complete block when treated with MBV, thereby confirming the specificity of the substrate Sox peptide. In the next step, we analyzed the effects of pUL97 + cyclin H cotransfection (Figure 9C). Signal intensities increased in a linear fashion starting after 20 min of the experiment. The linear increase of the curve between 20 and 70 min was used to calculate the relative pUL97 kinase activity per min, then normalized to pUL97 + vector sample, and protein amounts were normalized by Wb analysis. The results showed a 25% enhancement of pUL97 kinase activity under cyclin H coexpression, and even when considering moderate statistical significance, the finding provides initial evidence that pUL97–cyclin interaction stimulates pUL97 activity. Finally, another qSox-IVKA setting should address the question whether a pUL97 mutant lacking the IF2 region of cyclin H and T1 interaction (amino acids 231-280) shows an impairment of kinase activity under these conditions (Figure 9E,F). To this end, transiently expressed pUL97 Δ231-280-Flag and pUL97-Flag were compared, showing a decrease of kinase activity for the mutant Δ231-280 versus WT (Figure 9E; see relative pUL97 activity per min calculated for 20–60 min, normalized to pUL97-Flag in panel F). These findings in combination strongly support our notion that cyclin H binding has a positive impact on pUL97 kinase activity and that this regulatory property is a determinant of viral replication efficiency.

## 3. Discussion

Within the present study, we describe a many-layered panel of experimentation that addresses our focus on the functional importance of viral pUL97–cyclin interaction. A summarized view of the findings, obtained through the analysis of clinically relevant HCMV mutants and various methodological settings of recombinant expression, strongly supports our earlier data pointing to the functional significance of cyclin binding [10,20,22]. In vitro pUL97 kinase activity and the efficiency of viral replication demonstrate a clear enhancement mediated through pUL97–cyclin interaction, as measured by the independent testing systems, at least as far as cyclin H is concerned (Table 2). Clinically relevant virus mutants, carrying amino acid replacements in pUL97, with or without conferring drug resistance, behaved mostly like the WT in terms of viral replication kinetics, cyclin binding, and kinase activity (see Figure 1, Figure 2, Figure 3, Figure 4, Figure 7 and Figure 9). Importantly, however, deletion mutants in the cyclin IF2 region of pUL97, i.e., Δ231-280 or similar, resulted in defective or changed features of activity. Likewise, KO or modulated expression of cyclin H exerted a strong HCMV-specific, regulatory impact (see Figure 5, Figure 6, Figure 8, and Figure 9). These points add to our refined understanding of the highly conserved pUL97–cyclin interaction profiles among clinically relevant mutants, and the specific functional importance of human cyclin H for HCMV replication.

On a broader basis of discussion, the current findings illustrate at least three interesting aspects referring to pUL97–cyclin interaction: (i) These protein complexes represent very interesting examples of regulatory virus–host interplay; (ii) the increasing understanding of pUL97–complexes may open up new options of antiviral drug targeting strategies, in particular when considering the validated functional importance of cyclin H for HCMV replication; and (iii) the question arises whether this property of vCDK–cyclin interaction is a common phenomenon of herpesviruses.

As far as the first aspect is concerned, it should be emphasized that the regulatory interplay between HCMV replication and the CDK–cyclin machinery of host cells is a complex field that includes virus-induced G1/S phase arrest, pseudomitosis, and transcriptional reprogramming [11,12,15,54,55,56]. In this regard, the multifaceted regulatory capacity of pUL97 as a viral CDK ortholog resembles more the multiple cyclin-binding CDKs (such as the major cell-cycle-regulating CDKs 1, 2, and others) rather than the single cyclin-binding CDKs (such as the transcriptionally active CDKs 7, 9, and others) [25]. It is also striking that most of the viral and cellular pUL97 substrates are additionally phosphorylated by host CDK–cyclin complexes, supporting this concept of regulatory interplay through dual phosphorylation at the substrate level, in some cases at identical sites (e.g., lamin A/C S22; retinoblastoma protein T356, T373, S608, S612, S780, S788, S795, S807, S811, T821, T826; or SAMHD1 T592), in other cases at different or unknown sites [11,57]. Thus, HCMV pUL97 appears to hijack cellular functions associated with CDK and vCDK cyclin binding activity, to the benefit of viral replication.

Secondly, this key passage of virus–host interaction may be accessible as a target point for mechanistically new antiviral drugs. Very recently, MBV/LivtencityTM, the selective inhibitor of pUL97 [43], has been approved for the therapy of HCMV infection and disease refractory to standard treatment [58]. The mode of MBV inhibition acts as a classical ATP-competitive binder that blocks essential functions within the pUL97 kinase domain. In order to build upon this pUL97-directed antiviral strategy, and broaden the options arising from it, one might consider modes of inhibition that are independent from its kinase domain, i.e., acting in an ATP-noncompetitive manner [59]. Such an antiviral MoA might be achieved by the identification of small molecules that either block the pUL97 recognition of specific substrates or interfere with pUL97–cyclin binding. As far as the latter point is concerned, our initial screening experiments, using small molecules derived from the Prestwick Chemical Library^®^ of approved drugs, provided experimental evidence that inhibitors with the potential to block HCMV-specific protein interactions can display strong antiviral activity [60,61]. This strategy might similarly be adapted to the screening of sterical inhibitors of pUL97–cyclin assembly.

Finally, it should be considered whether cyclin interaction is a conserved property of other herpesviral vCDKs, or whether it is specific to HCMV. A related study strongly suggested that cyclin A interaction may be a conserved signature of β-herpesviral vCDKs [18]. This conclusion was drawn from quantitative proteomic analyses, which demonstrated that cyclin recruitment via the classical cyclin binding motif RXL is conserved for vCDKs of human herpesviruses 6 and 7 as well as rodent CMVs. Our group previously demonstrated that the pUL97-homologous vCDKs of additional β-herpesviruses, such as murine, rat, and rhesus monkey CMVs, also show cyclin B1 binding activity (M.M., 2016, 3.05, Session 4A, 41st Int. Herpesvirus Workshop, Madison, WI, USA). Although this finding has not been fully explored, our initial study of a γ-herpesviral vCDK provided further support of the notion that cyclin B1 and other types of human cyclins may form additional, so far undetected vCDK–cyclin complexes (M.M., Ma.S. et al., unpublished results).

In essence, the data of the present study underline the importance of host cyclin interaction for herpesviral vCDKs, especially HCMV pUL97, and illustrate the complexity and conservation of the interaction profiles for clinically relevant HCMVs. Specific focus was given to the pUL97–cyclin H interaction and the functional significance of this type of virus–host interaction was demonstrated from methodically different approaches. Subsequent interdisciplinary studies may validate the viral cyclin complexes as a putative antiviral target point, and may further address the fine-regulatory aspects of this important determinant of viral replication.

## 4. Materials and Methods

### 4.1. Cells and Viruses

Primary human foreskin fibroblasts (HFFs, C0045C, Thermo Fisher Scientific, Waltham, MA, USA) were cultured in minimal essential medium (MEM, 21090055, Thermo Fisher Scientific, Waltham, MA, USA) containing 10% FCS, 1x GlutaMAXTM (35050038, Thermo Fisher Scientific, Waltham, MA, USA), and 10 g/mL gentamicin (22185.03, SERVA, Heidelberg, Germany). Parental or recombinant HCMVs originating from strains TB40 or AD169 were used for infection experiments at a multiplicity of infection (MOI) of 0.5 or lower, as indicated for each experiment. Virus inocula were replaced with fresh growth medium after incubation at 37 °C for 90 min [10,16,39,53,62,63]. The origins of the clinical isolates used in this study for genetic marker experiments are given in Table 3.

### 4.2. Whole Genome Sequencing

The SureSelectXT target enrichment system for Illumina paired-end multiplexed sequencing (Agilent, Waldbronn, Germany) was used for sample preparation, according to manufacturer’s protocol (version C3, September 2019) [37]. In brief, one T175 cell culture flask containing HCMV-infected cells was harvested and DNA was extracted using a DNeasy Blood and Tissue Kit (Qiagen, Hilden, Germany). Then, 3 µg of DNA was sheared using a Bioruptor Plus (Diagenode SA, Liège, Belgium) for 30 cycles and 30/90 s on/off times, in a total volume of 100 µL. DNA fragments were purified using AMPure XP beads (Beckman Coulter GmbH, Krefeld, Germany) and DNA ends were repaired. In the next step, a dA-tail was added to the 3′ end of the DNA fragments and a paired-end adaptor was ligated to the DNA fragments. After a purification step using AMPure XP beads, the adaptor-ligated library was amplified via a 6-cycle PCR, again purified with AMPure XP beads and then stored at −20 °C. A biotinylated RNA bait library based on 201 complete HCMV genomes was utilized to enrich HCMV-specific DNA fragments (part number: 5191-6707, Agilent, Waldbronn, Germany) [64]. After amplification of the captured library with isolate-specific index primers, the paired-end samples were sequenced using NextSeq (Illumina, San Diego, CA, USA) at a read-length of 76 nt. Subsequently, reads were trimmed and aligned to the HCMV Merlin reference genome (NC_006273.2) using CLC workbench version 22.0. Annotations were extracted from HCMV Merlin and transferred to consensus sequences if a similarity of at least 96 % was given using SnapGene Vs 5.2.4.3.

### 4.3. Generation of Recombinant HCMVs

**Figure 10:**
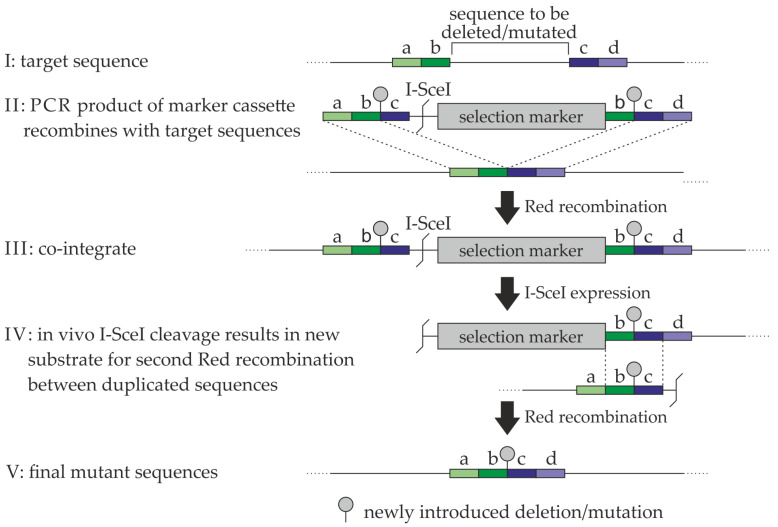
Schematic view of the generation of recombinant HCMVs. For a stepwise description of the procedure, see Results and Methods section. a–d, defined stretches of 50 nucleotides for primer design; adapted with permission from Tischer et al., 2010 [65]. © 2022, Springer Science+Business Media, LLC.

The tools and strategy of the two-step markerless Red recombination (summarized by Figure 10) were based on to earlier information provided by previous researchers [65]. BACmid TB40E IE2-YFP was used for the generation of resistance-conferring ORF-UL97 point mutations [38]. To this end, primers complementary to up- and downstream areas of the region to be deleted or exchanged within pUL97 were used to amplify a resistance cassette conferring kanamycin resistance (primers are listed in Table S2). Subsequent homologous recombination of the cassette with the target sequence led to deletion or exchange of the desired sequence. Positive clones were identified by the kanamycin-resistance marker and, after sequencing, were used for the second recombination step. Then, arabinose-dependent induction of the restriction enzyme I-SceI and cleavage of the DNA resulted in a second round of recombination, thereby again deleting the resistance cassette. The successful deletion of the desired sequence was again verified via sequencing (Figure 10, steps I–V). Recombinant viruses were reconstituted by transfection of HFF, using the Fugene transfection reagent according to the manufacturer’s protocol (Promega, Madison, WI, USA). The correctness of reconstituted viral DNA was again verified by sequencing. Regarding the HCMV mutant versions harboring single point mutations conferring GCV-resistance (A594V) or no-resistance mutation (P132L), shown in Figure 2C, viral mutants were generated from a bacterial artificial chromosome (BAC) pAL1111 cloned genome of strain Merlin using a selection cassette (kindly provided by Barry Slobedman, The University of Sydney, Australia) and a recombineering protocol described previously [52,66]. The Gaussia luciferase reporter gene was first integrated between US1 and the intergenic region between the US1 and US2 genes, followed by introduction of the mutations A594V and P132L to the pUL97 ORF. Reconstitution of HCMV mutants was performed by transfection of 0.8 × 106 MRC-5 cells with 800 ng BAC DNA using Lipofectamine 2000 reagent (Invitrogen, Karlsruhe, Germany) and OPTI-MEM I reduced serum medium (A4124801, Thermo Fisher Scientific, Waltham, MA, USA) according to manufacturers’ instructions [62]. Viral working stocks were titrated using standard plaque assay or IE1-specific immunostaining, and the UL97, UL54, and UL27 genes of all viral stocks were verified by sequencing analysis.

### 4.4. Generation of Cyclin KO Cell Lines

Specific guide RNAs (gRNAs) against cyclin B1, T1, or H were designed using Benchling Biology Software (2022), retrieved from https://benchling.com) and the scoring method of Hsu et al., 2013 [63]. Sequences, on-target, and off-target scores are listed in Table S3. CRISPR/Cas9 vectors were cloned according to Zhang laboratory protocol using lentiCRISPR v2; lentiCRISPR v2 was a gift from Feng Zhang (Addgene plasmid # 52961; http://n2t.net/addgene:52961, accessed on: 3 August 2021; RRID: Addgene_52961) [67]. 293T cells were transfected with lentiCRISPR v2, together with plasmids coding for the envelope protein G of vesicular stomatitis virus and HIV-1 gag/pol/rev, using Lipofectamine 2000 (Invitrogen, Karlsruhe, Germany). Lentiviral supernatants were harvested 48 h post-transfection, centrifuged for 5 min at 2000 rpm to remove cell debris, sterile filtered, and stored in aliquots at −80 °C, or used immediately to transduce young passage HFF cells. To this end, 8 × 10^4^ cells were seeded in 6-well plates. The following day, HFF cells were incubated with 1 mL of lentiviral supernatant supplemented with 1 mL MEM and 7.5 µg/mL polybrene (Sigma Aldrich, St. Louis, MO, USA) per well. The medium was removed 24 h after transduction and replaced by fresh medium. Selection was started the next day by adding 5 µg/mL puromycin to the medium. Selected cell populations were then used for subsequent experiments, as described previously [68]. In the case of cyclin H, KO resulted in completely defective cell division and therefore no cyclin H KO cell populations could be generated. For this reason, cells were subjected to a transient KO. Cells were transduced as described above using a cocktail of gRNAs A, B, C, E, and F, but were not selected. Instead, 2 days after transduction, KO cells and untreated control cells were used for infection experiments (see Section 4.1), and efficiency of KO was measured in parallel using Wb analysis (see Section 4.5).

### 4.5. Coimmunoprecipitation (CoIP), SDS-PAGE, and Western Blot (Wb) Analysis

For each sample, either a T75 or T175 flask of dense HFFs was used for infection at an MOI of approximately 0.5. The cells were harvested and lysed between 3 and 7 d.p.i. when a cytopathic effect (CPE) was detectable for approximately 80% of the cells. Coimmunoprecipitation (CoIP) was performed as described previously [23]. Input controls, immunoprecipitation (IP) and CoIP samples were subjected to standard SDS-PAGE and Wb analysis [69,70]. The antibodies used are listed in Section 4.6.

### 4.6. Antibodies

The following antibodies were used for this study [71]: mAb-UL97.01 (kindly provided by T. Lenac and S. Jonick; Department of Histology and Embryology, University of Rijeka, Croatia, used for Wb analysis), pAb-UL97 (kindly provided by D. M. Coen, Harvard Medical School, Boston, used for Wb analysis), mAb-β-Actin (A5441, Sigma Aldrich, used for Wb analysis), mAb-Cyclin B1 (sc-245, Santa Cruz, used for Wb analysis), pAb-Cyclin B1 (AF6000, R&D Systems, used for IP and Wb analysis), pAb-Cyclin T1 (ab226851, abcam, used for IP and Wb analysis), pAb-Cyclin H (LS-C331195, LS Bio, used for IP and Wb analysis), and Fc chicken (003-000-008, Jackson ImmunoResearch Laboratories Inc., West Grove, PA, USA).

### 4.7. Quantitative Polymerase Chain Reaction (qPCR)

A total of 2.25 × 10^5^ HFFs were seeded in 12-well plates and infected the next day with 1 × 10^5^ HCMV genome equivalents, if not stated otherwise, for 90 min at 37 °C. Samples of viral supernatant were taken at the indicated time points and replication kinetics were analyzed via qPCR by the amplification of a specific region in the IE1 gene locus (ORF-UL123, exon 4), using a FAM/TAMRA-labeled probe as described previously [10,63,69].

### 4.8. HCMV Fluorescence-Based Replication Assay

HCMV GFP-based replication assays were performed as described previously [49,72]. In brief, 2.25 × 10^5^ HFFs were cultivated in 12-well plates for the infection with parental HCMV AD169-GFP or UL97 deletion mutants at an MOI of 0.25 GFP-FU/cell. Cells were lysed at indicated time points, and total lysates were subjected to automated GFP quantitation using a Victor 1420 multilabel counter (Perkin-Elmer, Waltham, MA, USA). Mean values and SDs of quadruplicate determinations are given (infections in duplicate, GFP measurements in duplicate). For a quantitative measurement of HCMV IE2-YFP-positive cells, 1.35 × 10^4^ HFFs were seeded in 96-well plates, infected at an MOI of 0.01, and cells were counted at given time points using ImageXpress Pico (Molecular Devices LLC, San Jose, CA, USA) and the system-integrated cell count assay.

### 4.9. Quantitative Sox Peptide-Based In Vitro Kinase Assay (qSox-IVKA)

For the quantitative measurement of in vitro pUL97 kinase activity, a Sox-based peptide was used according to technical principles described elsewhere (AQT0258, Assayquant Technologies Inc., Marlboro, MA, USA) [46,47]. For this purpose, 5 × 106 cells were seeded in 10-cm^2^ dishes and transfected with plasmids coding for pUL97-Flag, cyclin H-HA, or empty pcDNA3.1 vector as a control, using polyethylenimine-DNA complexes (Sigma Aldrich, St. Louis, MO, USA) as described previously [73]. Cells were harvested, and pUL97 or empty vector as negative control immunoprecipitated using pUL97-specific antibodies with 25 µL Dynabeads^®^ (10002D, Thermo Fisher Scientific, Waltham, MA, USA). Precipitated samples were eluted in 50 µL enzyme buffer (20 mM HEPES, pH 7.5, 0.1% Brij-35, 5% glycerol, 1 mg/mL bovine serum albumin) and then used for the in vitro kinase assay. At this point, 10 µL of precipitate was added to 40 µL of the kinase reaction mix (final mix: 54 mM HEPES, pH 7.5, 1.0 mM DTT, 1.0 mM ATP, 0.012% Brij-35, 0.52 mM EGTA, 1% glycerol, 0.2 mg/mL BSA, 10 mM MgCl2, 15 μM PhosphoSenS^®^ cysteine-Sox kinase sensor peptide). Where required, 25 µL of precipitate was used for Wb normalization in duplicate. Optionally, 5 µM MBV or an equal amount of DMSO was added for specificity control. Reactions were run in Corning NBS 96-well half area plates (#3992, Corning, Glendale, AZ, USA) at 30 °C for approximately 80 min, using a Victor 1420 multilabel counter (Ex 355/Em 480, Perkin-Elmer, Waltham, MA, USA). Measurements were taken in duplicate and the empty vector control signal was subtracted from the pUL97 signal intensity to obtain kinase specific activity.

## Figures and Tables

**Table 1:** Details of HCMV Ulm isolates and ORF-UL97 primary sequences.

Clinical Isolate	Mode of Viral Reproduction ^a^	Numbers of Passages	GenBank Accession Number
E16662	cell-bound mainly (cell-free 5.03 × 10^6^ copies/µL)	<5	ON119190
E18800	cell-bound mainly (cell-free 1.10 × 10^6^ copies/µL)	<5	ON119191
E20744	cell-bound mainly (cell-free 2.77 × 10^6^ copies/µL)	<5	ON119192
E20749	cell-bound mainly (cell-free 3.99 × 10^5^ copies/µL)	<5	ON119193
E9045	cell-bound mainly (cell-free 4.11 × 10^6^ copies/µL)	<5	ON119194
E9361	cell-bound mainly (cell-free 2.74 × 10^6^ copies/µL)	<5	ON119195
E31354	cell-bound mainly (cell-free 9.38 × 10^5^ copies/µL)	<5	ON119196
E83151	cell-bound mainly (cell-free 1.86 × 10^6^ copies/µL)	<5	ON119197
E79446	cell-bound mainly (cell-free 1.11 × 10^6^ copies/µL)	<5	ON119198
E29747	cell-bound mainly (cell-free 2.36 × 10^6^ copies/µL)	<5	ON119199

^a^ Virus infectivity was determined by infection experiments and plaque formation, using primary infected cell-media supernatants; these experiments showed that cell-free infectious titers were generally low for all these isolates (viral genomic copy numbers detected by HCMV-specific qPCR, mean values of triplicate determinations are given), i.e., the main infectivity remained cell-bound.

**Table 2:** Viral characteristics relating to pUL97-cyclin interaction.

	Mutant pUL97	Viral Replication Efficiency	Replication in Cyclin KO Cells	CoIP pUL97-Cyclin	Activity of pUL97	Cyclin Type with Main Impact on pUL97 and HCMV Replication	Source/Origin of Virus Strains and Recombinants
Laboratory strains (AD169, TB40, Merlin)	no	normal	cycB1/T1: normal cycH KO: impaired	cyclins B1/T1/H	normal	cyclin H	[16,38,44,45,48,49,50,51,52,53]
Deletion mutant pUL97 Δ231-280	yes	defective	n.d.	cyclin B1 only	drastic changes	cyclin H	[10]
Drug resistance pUL97 point mutants	yes	Normal	n.d.	cyclins B1/T1/H	t.b.d.	cyclin H	[present study]

n.d., not determined.

**Table 3:** Origins of clinical isolates used in this study.

Isolate	Collected by	Collection Date	Country	Isolation Source	Host
E16662	T. Stamminger lab	28 January 1997	Germany	human clinical specimens	H. sapiens
E18800	T. Stamminger lab	7 January 1999	Germany	human clinical specimens	H. sapiens
E20744	T. Stamminger lab	5 March 1999	Germany	human clinical specimens	H. sapiens
E20749	T. Stamminger lab	15 March 1999	Germany	human clinical specimens	H. sapiens
E9045	T. Stamminger lab	7 December 2000	Germany	human clinical specimens	H. sapiens
E9361	T. Stamminger lab	7 December 2000	Germany	human clinical specimens	H. sapiens
E31354	T. Stamminger lab	21 August 2003	Germany	human clinical specimens	H. sapiens
E83151	T. Stamminger lab	25 November 2014	Germany	human clinical specimens	H. sapiens
E79446	T. Stamminger lab	14 October 2014	Germany	human clinical specimens	H. sapiens
E29747	T. Stamminger lab	15 June 2011	Germany	human clinical specimens	H. sapiens

## Data Availability

The data presented in this study are available on request from the corresponding author.

## Supplementary Materials

The following supporting information can be downloaded at: https://www.mdpi.com/article/10.3390/ijms231911814/s1.

## Abbreviations

ATP	adenosine triphosphate
CAK	CDK-activating kinase
CDK	cyclin-dependent kinase
cCMV	congenital cytomegalovirus infection
CoIP	coimmunoprecipitation
DMEM	Dulbecco’s modified Eagle’s medium
DMSO	dimethyl sulfoxide
d p.i.	days post infection
GCV	ganciclovir
GFP	green fluorescent protein
gRNA	guide RNA
HCMV	human cytomegalovirus
IF	interface
HFF	human foreskin fibroblast
IVKA	in vitro kinase assay
KO	knock-out
MBV	maribavir
MOI	multiplicity of infection
NLS	nuclear localization signal
ORF	open reading frame
qPCR	quantitative polymerase chain reaction
-R	-resistant
Rb	retinoblastoma protein
vCDK	viral cyclin-dependent kinase ortholog
WGS	whole genome sequencing
Wb	Western blot
WT	wild-type
YFP	yellow fluorescent protein
