# Supplementary material for: Highly Conserved Interaction Profiles between Clinically Relevant Mutants of the Cytomegalovirus CDK-like Kinase pUL97 and Human Cyclins: Functional Significance of Cyclin H
Source: Int J Mol Sci. 2022 Oct 5;23(19):11814. doi: 10.3390/ijms231911814 (PMC9569496; doi:10.3390/ijms231911814)
Supplement: Supplementary file 1 [file ijms-23-11814-s001.zip › ijms-1903130-supplementary.pdf]

Supplementary Material

Highly conserved interaction profiles between clinically relevant mutants of the cytomegalovirus CDK-like kinase pUL97 and human cyclins: functional significance of cyclin H

Martin Schütz[1,*], Regina Müller[1], Eileen Socher[1,2,3], Christina Wangen[1], Diana Wong[4], Florian Full[5], Emanuel Wyler[6], Myriam Scherer[7], Thomas Stamminger[7], Sunwen Chou[8,9], William D. Rawlinson[4], Stuart T. Hamilton[4,] , Heinrich Sticht[2,*] & Manfred Marschall[1,*]

[1] Institute for Clinical and Molecular Virology, Friedrich-Alexander-Universität Erlangen-Nürnberg (FAU), Schlossgarten 4, 91054 Erlangen, Germany

[2] Division of Bioinformatics, Institute of Biochemistry, FAU Erlangen-Nürnberg, Germany

[3] Functional and Clinical Anatomy, Institute of Anatomy, FAU Erlangen-Nürnberg, Germany

[4] Serology and Virology Division, NSW Health Pathology Microbiology, Prince of Wales Hospital, Schools of Women's and Children's Health, Medicine and Biotechnology and Biomolecular Sciences, University of New South Wales, Sydney, Australia

[5] Department of Medical Microbiology, Virology and Hygiene, Institute for Virology, Freiburg, Germany

[6] Max-Delbrück-Center for Molecular Medicine (MDC), Berlin, Germany

[7] Institute for Virology, Ulm University Medical Center, Ulm, Germany

[8] Division of Infectious Diseases, Oregon Health and Science University, Portland, Oregon, USA

[9] Department of Veterans Affairs Medical Center, Portland, Oregon, USA

*Corresponding authors: manfred.marschall@fau.de; heinrich.sticht@fau.de; martin.schuetz@uk-erlangen.de

Supplementary Tables.

Table S1. List of 473 ORF-UL97 GenBank accession numbers.

YP_081544.1	AEF33987.1	AKI22520.1	QZX46450.1	APG57625.1	AKI17170.1	AJZ68912.1
AAS48981.1	AEF33986.1	AKI22352.1	QZX46283.1	APG57456.1	AKI16669.1	AJZ68911.1
ASY06521.1	AEF33985.1	AKI22187.1	QZX46115.1	APG57286.1	AKI16506.1	AJZ68910.1
ASY06353.1	AEF33984.1	AKI21686.1	QZX45948.1	AGT36515.1	AKI16339.1	AJZ68909.1
ASY06184.1	AEF33979.1	AKI21520.1	QUP52178.1	ACS32399.1	AKI16170.1	AJZ68908.1
ASY06016.1	AEF33975.1	AKI21021.1	QTT59577.1	APA46333.1	AKI16004.1	AJZ68907.1
AAV49340.1	AEF33973.1	AKI20853.1	QTT59409.1	APA45708.1	AKI15838.1	AJZ68906.1
AAV49339.1	AEF33968.1	AKI20014.1	QTT59241.1	APA45101.1	AKI15505.1	BAF64756.1
AAV49338.1	AEF33956.1	AKI19514.1	QTT59075.1	AND82017.1	AKI15337.1	CAC42715.1
AAV49337.1	ABV04334.1	AKI18675.1	QTT58907.1	AND81688.1	AKI15170.1	ALT14842.1
AAV49336.1	ABV04333.1	AKI18508.1	QTT58739.1	AND81523.1	AKI15002.1	ALT14677.1
AAV49335.1	AHV84044.1	AKI18339.1	QTT58571.1	AKI26591.1	AKI14330.1	ALK03053.1
AAV49334.1	YP_081544.1	AKI17002.1	QTF98549.1	AKI26422.1	AKI14162.1	AHJ85175.1
AAV49332.1	AVT50513.1	AKI16837.1	QTF98383.1	AKI26085.1	AKI13996.1	AHJ84668.1
AAV49329.1	AVT50344.1	AKI15671.1	QPZ45359.1	AKI25917.1	AKI13830.1	AHJ82480.1
AAV49328.1	AQN73919.1	AKI14834.1	QPZ45196.1	AKI25749.1	AKI13494.1	AHJ86183.1
AAV49327.1	AQN73413.1	AKI14667.1	QPZ45031.1	AKI25581.1	AKI13325.1	AKI23355.1
AAV49326.1	AQN71730.1	AKI14499.1	QPZ44867.1	AKI25412.1	AKI13157.1	AKI22855.1
AAV49325.1	AQN69707.1	AKI13662.1	QPZ44704.1	AKI25242.1	AKI12991.1	AEF33957.1
QNT12642.1	AMJ54958.1	AKI12659.1	QPI35515.1	AKI24397.1	AKI12823.1	AEF33955.1
QGH81923.1	AMJ54625.1	AKI12326.1	QPI35345.1	AKI24227.1	AKI12492.1	AEF33954.1
QHB20536.1	AMJ53955.1	AKI11329.1	QPI35176.1	AKI23890.1	AKI12160.1	AEF33953.1
ABV71619.1	AMJ53456.1	AKI10996.1	QPI35006.1	AMD82420.1	AKI11992.1	AAA16790.1
APA46116.1	AMJ52789.1	AKI10829.1	BCM78255.1	AMO64771.1	AKI11825.1	UBQ34163.1
AAV49333.1	AIC80516.1	AKI09486.1	QIA46247.1	AMO64436.1	AKI11661.1	QZX46957.1
AAV49331.1	AIC80348.1	AKI09150.1	QIA46078.1	AHJ83825.1	AKI11497.1	QZX46789.1
AAV49330.1	ACZ72839.1	AKI08982.1	QIA45911.1	AHJ83658.1	AKI11162.1	QZX46620.1
AAV49324.1	APB97551.1	AKI08814.1	QIA45741.1	AHJ83491.1	AKI10661.1	AMJ54123.1
AAV49323.1	APB97382.1	AKI07810.1	QIA45572.1	AHJ83153.1	AKI10494.1	AMJ53789.1
P16788.1	APA45350.1	AJZ68925.1	QIA45403.1	AHJ82984.1	AKI10327.1	AMJ53624.1
Q6SW46.1	AJY57929.1	AJZ68924.1	QIA45234.1	AHJ82815.1	AKI10160.1	AMJ53289.1
Q68101.1	AJY57763.1	AJZ68922.1	QIA45066.1	AFR56417.1	AKI09991.1	AMJ53122.1
AAL10779.1	AJY57596.1	AJZ68921.1	QIA44896.1	AFR56250.1	AKI09822.1	AMJ52956.1
AAL10777.1	AJY57429.1	AJZ68918.1	QIA44728.1	AFR55749.1	AKI09655.1	AMJ52623.1
AAL10775.1	AJY57262.1	AJZ68917.1	QBF76518.1	AFR55580.1	AKI09319.1	AIC80180.1
AAL10772.1	AJY57095.1	AJZ68916.1	AZV24374.1	AFR55415.1	AKI08645.1	APG57963.1
AAL10771.1	AJY56928.1	AII80189.1	AZV24204.1	AFR55246.1	AKI08478.1	AKI19177.1
AAL10767.1	AJY56761.1	AII80022.1	AZV24034.1	AFR55078.1	AKI08312.1	AKI19010.1
AAL10766.1	AJY56594.1	AII79863.1	QBK84323.1	AFR54914.1	AKI08145.1	AKI18843.1
AAL10764.1	AJY56427.1	AII79698.1	ARX80476.1	AFR54747.1	AKI07978.1	AKI18172.1
AAL10790.1	AJY56260.1	AII79530.1	ARX80308.1	AFR54587.1	AKI07642.1	AKI18006.1
AAL10789.1	AJY56093.1	AHB19899.1	AQN73750.1	AHJ86015.1	AHB20064.1	AKI17839.1

AAL10788.1	AJY55926.1	AGL96685.1	AQN73581.1	AHJ85848.1	AHB19733.1	AKI17673.1
AAL10787.1	AJY55759.1	AAR31648.1	AQN73245.1	AHJ85681.1	AHB19566.1	AKI17506.1
AAL10786.1	AJY55592.1	ACT81767.1	AQN73077.1	AHJ85513.1	AHB19398.1	AKI17339.1
AAL10785.1	AJY55425.1	ACS93428.1	AQN72907.1	AHJ85344.1	AGT36386.1	ACF74517.1
AAL10784.1	AJY55258.1	ACS92186.1	AQN72738.1	AHJ85005.1	AGQ47315.1	AZB79970.1
AAL10783.1	AJY55091.1	BAJ06835.1	AQN72570.1	AHJ84837.1	ACZ80337.1	ADV04413.1
AAL10782.1	AJY54924.1	AEF33983.1	AQN72401.1	AHJ84499.1	ACZ80172.1	ABA26301.1
AAL10781.1	AJY54757.1	AEF33982.1	AQN72233.1	AHJ84331.1	ACZ80007.1	ABA26313.1
AAL10780.1	ADD39142.1	AEF33981.1	AQN72064.1	AHJ84163.1	ACZ79843.1	QHX40593.1
AAL10778.1	AND81852.1	AEF33980.1	AQN71897.1	AHJ82143.1	ACU83746.1	QHX40112.1
AAL10776.1	AKI24903.1	AEF33978.1	AQN71560.1	AHJ81975.1	ACT81932.1	CAA35333.1
AAL10774.1	AMO64939.1	AEF33977.1	AQN71391.1	ALN67084.1	ACS92021.1	AKI24565.1
AAL10773.1	AMO64604.1	AEF33976.1	AQN71222.1	ALL26239.1	ACM48074.1	AKI24058.1
AAL10770.1	AHJ83995.1	AEF33974.1	AQN71053.1	AKI23522.1	ACL51165.1	AKI23722.1
AAL10769.1	AHJ83322.1	AEF33972.1	AQN70884.1	AKI23190.1	DAA00194.1	AKI19847.1
AAL10768.1	AHJ82649.1	AEF33971.1	AQN70715.1	AKI23023.1	AAA16789.1	AKI19679.1
AAL10765.1	AFR56083.1	AEF33970.1	AQN70546.1	AKI22687.1	APG57795.1	AKI19346.1
AAL10763.1	AFR55915.1	AEF33969.1	AQN70380.1	AKI22021.1	APA46260.1	AMJ54790.1
AAV85476.1	ADE88088.1	AEF33967.1	AQN70213.1	AKI21853.1	APA45838.1	AMJ54457.1
ALK03055.1	ALT16318.1	AEF33966.1	AQN70043.1	AKI21356.1	APA45592.1	AMJ54290.1
ALK03054.1	ALT16153.1	AEF33965.1	AQN69874.1	AKI21188.1	APA45474.1	AEF33960.1
ALK03052.1	ALT15997.1	AEF33964.1	AQN69539.1	AKI20685.1	APA45223.1	AEF33959.1
AJZ68923.1	ALT15832.1	AEF33963.1	AQN69370.1	AKI20517.1	AKI26254.1	AEF33958.1
AJZ68920.1	ALT15667.1	AEF33962.1	ATP76347.1	AKI20350.1	AKI25072.1	ALT15172.1
AJZ68919.1	ALT15502.1	AEF33961.1	AMJ55123.1	AKI20182.1	AKI24734.1	ALT15007.1
AJZ68915.1	ALT15337.1	AJZ68913.1	AJZ68914.1			

Table S2. Oligonucleotide primers used for BACmid recombinations.

H411Y-for	CACGGCCACGGGCTGCTGTCTGCTGCACAACGTCACGGTATATCGACGTTTCCACACAGACATTA GGGATAACAGGGTAATCGATTT
H411Y-rev	TCCACTGGTCGTGATGAAACATGTCTGTGTGGAAACGTCGATATACCGTGACGTTGTGCAGCAGC CAGTGTTACAACCAATTAACC
T409M-for	TCTGCTCACGGCCACGGGCTGCTGTCTGCTGCACAACGTCATGGTACATCGACGTTTCCACACTA GGGATAACAGGGTAATCGATTT
T409M-rev	GGTCGTGATGAAACATGTCTGTGTGGAAACGTCGATGTACCGTGACGTTGTGCAGCAGACAGCGC CAGTGTTACAACCAATTAACC
F342S-for	GGACATGAGCGACGAGAGCTACCGCCTGGGCCAGGGCTCCTCCGGCGAGGTCTGGCCGCTCGATA GGGATAACAGGGTAATCGATTT
F342S-rev	CCTTGACCACGCGATAGCGATCGAGCGGCCAGACCTCGCCGGAGGAGCCCTGGCCCAGGCGGTGC CAGTGTTACAACCAATTAACC
L595S-for	GGCGTTGCTCTTTAAGCACGCCGGCGCGGCCTGCCGCGCGTCGGAGAACGGCAAGCTCACGCATA GGGATAACAGGGTAATCGATTT
L595S-rev	GCAGACAGGCGTCGGAGCAGTGCGTGAGCTTGCCGTTCTCCGACGCGCGGCAGGCCGCGCCGGGC CAGTGTTACAACCAATTAACC
Del.599-600-for	TAAGCACGCCGGCGCGGCCTGCCGCGCGTTGGAGAACGGCACGCACTGCTCCGACGCCTGTAGGG ATAACAGGGTAATCGATTT
Del.599-600-rev	GCGCCGCCAGAATGAGCAGACAGGCGTCGGAGCAGTGCGTGCCGTTCTCCAACGCGCGGCGCCAG TGTTACAACCAATTAACC
H469V-for	TGATATTACACCCATGAACGTGCTCATCGACGTGAACCCGGTCAACCCCAGCGAGATCGTGCGTA GGGATAACAGGGTAATCGATTT
H469-rev	TGTAATCGCACAGCGCGGCGCGCACGATCTCGCTGGGGTTGACCGGGTTCACGTCGATGAGCAGC CAGTGTTACAACCAATTAACC
K359Q-for	CTGGCCGCTCGATCGCTATCGCGTGGTCAAGGTGGCGCGTCAGCACAGCGAGACGGTGCTCACTA GGGATAACAGGGTAATCGATTT
K359Q-rev	TCAGGCCCGACATCCAGACCGTGAGCACCGTCTCGCTGTGCTGACGCGCCACCTTGACCACGCGC CAGTGTTACAACCAATTAACC
L397R-for	GCCGCCGTCGCTGGTGGGCACGGGCGTGCACCGCGGTCTGCGCACGGCCACGGGCTGCTGTCTTA GGGATAACAGGGTAATCGATTT
L397R-rev	GTACCGTGACGTTGTGCAGCAGACAGCAGCCCGTGGCCGTGCGCAGACCGCGGTGCACGCCCGGC CAGTGTTACAACCAATTAACC
M460V-for	CAATCACCAGTGTCGTGTATGCCACTTTGATATTACACCCGTGAACGTGCTCATCGACGTGAATA GGGATAACAGGGTAATCGATTT
M460V-rev	TCTCGCTGGGGTTGTGCGGGTTCACGTCGATGAGCACGTTCACGGGTGTAATATCAAAGTGGCGC CAGTGTTACAACCAATTAACC
C480F-for	GAACCCGCACAACCCCAGCGAGATCGTGCGCGCCGCGCTGTTCGATTACAGCCTCAGCGAGCCTA GGGATAACAGGGTAATCGATTT
C480F-rev	GCTCGTTGTAATCCGGATAGGGCTCGCTGAGGCTGTAATCGAACAGCGCGGCGCGCACGATCTGC CAGTGTTACAACCAATTAACC
H520Q-for	CCGCATCCCCAACTGCTCGCACCGTCTGCGCGAATGTTACCAACCTGCTTTCCGACCCATGCCTA GGGATAACAGGGTAATCGATTT
H520Q-rev	AGATGAGCAGCTTCTGCAGCGGCATGGGTCGGAAAGCAGGTTGGTAACATTCGCGCAGACGGTGC CAGTGTTACAACCAATTAACC
C592G-for	GGGTACGGAGGCGTTGCTCTTTAAGCACGCCGGCGCGGCCGGCCGCGCGTTGGAGAACGGCAATA GGGATAACAGGGTAATCGATTT
C592G-rev	CGTCGGAGCAGTGCGTGAGCTTGCCGTTCTCCAACGCGCGGCCGGCCGCGCCGGCGTGCTTAAGC CAGTGTTACAACCAATTAACC
E596G-for	GTTGCTCTTTAAGCACGCCGGCGCGGCCTGCCGCGCGTTGGGGAACGGCAAGCTCACGCACTGTA GGGATAACAGGGTAATCGATTT
E596G-rev	TGAGCAGACAGGCGTCGGAGCAGTGCGTGAGCTTGCCGTTCCCCAACGCGCGGCAGGCCGCGCGC CAGTGTTACAACCAATTAACC

C603W-for	CGCGGCCTGCCGCGCGTTGGAGAACGGCAAGCTCACGCACTGGTCCGACGCCTGTCTGCTCATTA GGGATAACAGGGTAATCGATTT
C603W-rev	AGCTCATTTGCGCCGCCAGAATGAGCAGACAGGCGTCGGACCAGTGCGTGAGCTTGCCGTTCTGC CAGTGTTACAACCAATTAACC
US1-for	CGCATGGGAAGGCTGGACACGCCGACCGAGAGGTCACCGAGCCCGGACGCCATCCTGTGACGGAA GATCACTTCG
USIR-rev	GGAGCGGAGGAGGTGACCTGGCTGCTTAATGACAGCGACGGAGAGGAAGAAGAGCTGAGGTTCTT ATGGCTCTTG
US1-for	CCGCATGGGAAGGCTGGACACGCCGACCGAGAGGTCACCGAGCCCGGACGCCATCTGCTTCGCGA TGTACGGGCCAGATATA
US1-IR-rev	ACCGCGGAGCGGAGGAGGTGACCTGGCTGCTTAATGACAGCGACGGAGAGGAAGAAGAGATTGCA GCACAGAAAAGCATCTTAC
UL97-for	GGTCTGGACGAGGTGCGCATGGGTACGGAGGCGTTGCTCTTTAAGCACGCCGGCGCGGCCTGCCG CGCGTTGGAGAACCCTGTGACGGAAGATCACTTCG
UL97-rev	CGCCCAGGAGACAGGCGCCGTAGCTCATTTGCGCCGCCAGAATGAGCAGACAGGCGTCGGAGCAG TGCGTGAGCTTGCCCTGAGGTTCTTATGGCTCTTG
UL97-for	GATGCGTGACGGAGAAAAAGAGGACGCGGCTTCGGACAAGGAGAACCAGCGTCGGCCCGTGGTGC CGTCCACGTCCCTGTGACGGAAGATCACTTCG
UL97-rev	GAACGACCACATGGCCGAGGTTTCGCGGCAGCGCAAGCCGTGGTAACCGTCGCCGCTGGCGGCGC TGCCGCGAGACCTGAGGTTCTTATGGCTCTTG
A594V	CTTTAAGCACGCCGGCGCGGCCTGCCGCGTGTTGGAGAACGGCAAGCTCACGCACTGCTCCGACG CCTGTCTGCTCATTC
P132L	GCGGCTTCGGACAAGGAGAACCAGCGTCGGCCCGTGGTGCTGTCCACGTCGTCTCGCGGCAGCGC CGCCAGCGGCGACGGT
236-275 For	AGAAAACGACGTGGAGCTGCGCGCGGAAAGTCAGGACAGCAACGACCAGATCATCACCACTAGGG ATAACAGGGTAATCGATTT
236-275 Rev	ACGTAAGGCCGCGGATGGACGTGGTGATGATCTGGTCGTTGCTGTCCTGACTTTCCGCGCGCCAG TGTTACAACCAATTAACC
241-270 For	GCTGCGCGCGGAAAGTCAGGACAGCGCCGTGGCATCGGGCCATTGCACCTGTTCCAACGATAGGG ATAACAGGGTAATCGATTT
241-270 Rev	TGGACGTGGTGATGATCTGGTCGTTGGAACAGGTGCAATGGCCCGATGCCACGGCGCTGTGCCAG TGTTACAACCAATTAACC
246-265-For	TCAGGACAGCGCCGTGGCATCGGGCCCGGGCCGCATTCCGTCACACGACGACGTGCATTGTAGGG ATAACAGGGTAATCGATTT
246-265-Rev	TCTGGTCGTTGGAACAGGTGCAATGCACGTCGTCGTGTGACGGAATGCGGCCCGGGCCCGGCCAG TGTTACAACCAATTAACC
251-260 For	GGCATCGGGCCCGGGCCGCATTCCGCAGCCGCTCAGCGGTGAGGCCGACTCCACGTCACATAGGG ATAACAGGGTAATCGATTT
251-260 Rev	AGGTGCAATGCACGTCGTCGTGTGACGTGGAGTCGGCCTCACCGCTGAGCGGCTGCGGAAGCCAG TGTTACAACCAATTAACC

Table S3. Sequences, primers, target exon, on-target and off-target scores for CRISPR/Cas9 KO.

Name	Sequence	Primer	Exon	On-target	Off-target
Cyclin B1 (A)-For<br>Cyclin B1 (A)-Rev	AGGCGCAAAGCGCGT TCCTA	CACCGAGGCGCAAAGCGC GTTCCTA<br>AAACTAGGAACGCGCTTT GCGCCTC	2	47.3	96.0
Cyclin B1 (B)-For<br>Cyclin B1 (B)-Rev	CCTAATTGACTGGCT AGTAC	CACCGCCTAATTGACTGG CTAGTAC<br>AAACGTACTAGCCAGTCA ATTAGGC	5	50.0	85.8

Cyclin B1 (C)-For<br>Cyclin B1 (C)-Rev	CCATGGCGCTCCGAGTCACC	CACCGCCATGGCGCTCCGAGTCACC<br>AAACGGTGACTCGGAGCGCCATGGC	1	46.5	85.6
Cyclin T1 (A)-For<br>Cyclin T1 (A)-Rev	CCACGCCAAAACGACGGGAT	CACCGCCACGCCAAAACGACGGGAT<br>AAACATCCCGTCGTTTTGGCGTGGC	1	62.6	95.9
Cyclin T1 (B)-For<br>Cyclin T1 (B)-Rev	AGAACTTTCTTATCGCCAGC	CACCGAGAACTTTCTTATCGCCAGC<br>AAACGCTGGCGATAAGAAAGTTCTC	1	55.7	82.1
Cyclin T1 (C)-For<br>Cyclin T1 (C)-Rev	GTTTCTAGCAGCTAAAGTGG	CACCGGTTTCTAGCAGCTAAAGTGG<br>AAACCCACTTTAGCTGCTAGAAACC	3	69.3	70.7
Cyclin H (A)-For<br>Cyclin H (A)-Rev	GCCGCTTCTGACTACTGTTG	CACCGGCCGCTTCTGACTACTGTTG<br>AAACCAACAGTAGTCAGAAGCGGCC	1	64.5	82.9
Cyclin H (B)-For<br>Cyclin H (B)-Rev	GTCCAAGAGGACTCTCCCGG	CACCGGTCCAAGAGGACTCTCCCGG<br>AAACCCGGGAGAGTCCTCTTGGACC	4	73.4	82.5
Cyclin H (C)-For<br>Cyclin H (C)-Rev	ATTCTCCAATATGGGATAGC	CACCGATTCTCCAATATGGGATAGC<br>AAACGCTATCCCATATTGGAGAATC	5	60.5	79.0
Cyclin H (E)-For<br>Cyclin H (E)-Rev	CCGGGAGAGTCCTCTTGGAC	CACCGCCGGGAGAGTCCTCTTGGAC<br>AAACGTCCAAGAGGACTCTCCCGGC	4	52.8	44.0
Cyclin H (F)-For<br>Cyclin H (F)-Rev	CAGTAATGGAATATCACCCC	CACCGCAGTAATGGAATATCACCCC<br>AAACGGGGTGATATTCCATTACTGC	3	62.5	43.1
